# Reconstructing the Three-Dimensional GABAergic Microcircuit of the Striatum

**DOI:** 10.1371/journal.pcbi.1001011

**Published:** 2010-11-24

**Authors:** Mark D. Humphries, Ric Wood, Kevin Gurney

**Affiliations:** 1 Adaptive Behaviour Research Group, Department of Psychology, University of Sheffield, Sheffield, United Kingdom; 2 Group for Neural Theory, Department d'Etudes Cognitives, Ecole Normale Superieure, Paris, France; University College London, United Kingdom

## Abstract

A system's wiring constrains its dynamics, yet modelling of neural structures often overlooks the specific networks formed by their neurons. We developed an approach for constructing anatomically realistic networks and reconstructed the GABAergic microcircuit formed by the medium spiny neurons (MSNs) and fast-spiking interneurons (FSIs) of the adult rat striatum. We grew dendrite and axon models for these neurons and extracted probabilities for the presence of these neurites as a function of distance from the soma. From these, we found the probabilities of intersection between the neurites of two neurons given their inter-somatic distance, and used these to construct three-dimensional striatal networks. The MSN dendrite models predicted that half of all dendritic spines are within 100µm of the soma. The constructed networks predict distributions of gap junctions between FSI dendrites, synaptic contacts between MSNs, and synaptic inputs from FSIs to MSNs that are consistent with current estimates. The models predict that to achieve this, FSIs should be at most 1% of the striatal population. They also show that the striatum is sparsely connected: FSI-MSN and MSN-MSN contacts respectively form 7% and 1.7% of all possible connections. The models predict two striking network properties: the dominant GABAergic input to a MSN arises from neurons with somas at the edge of its dendritic field; and FSIs are inter-connected on two different spatial scales: locally by gap junctions and distally by synapses. We show that both properties influence striatal dynamics: the most potent inhibition of a MSN arises from a region of striatum at the edge of its dendritic field; and the combination of local gap junction and distal synaptic networks between FSIs sets a robust input-output regime for the MSN population. Our models thus intimately link striatal micro-anatomy to its dynamics, providing a biologically grounded platform for further study.

## Author Summary

The brain has an immensely complex wiring diagram, but few computational models of brain regions attempt accurate renditions of the wiring between neurons. Consequently, these models' dynamics may not accurately reflect those of the region. Key barriers here are the difficulty of reconstructing such networks and the paucity of critical data on neuron morphology. We demonstrate an approach that gets around these problems by using the available data to construct prototype neuron morphologies, and uses these to estimate how the probability of a connection between two neurons changes as we change the distance between them. With these in hand, we constructed artificial three-dimensional networks of the rat striatum and find that the connection distributions agree well with current estimates from anatomical studies. Our networks show features and dynamical implications of striatal wiring that would be difficult to intuit: the dominant input to the striatal projection neuron arises from other neurons just at the edge of its dendrites, and the main inhibitory interneurons are coupled locally by electrical connections and more distally by chemical synapses. Together, these properties set a unique state for the input-output computations of the striatum.

## Introduction

The mammalian brain is a vastly complex structure at every level of description. Faced with the sheer breadth of neuron and receptor types, many researchers are abandoning attempts to intuit the ‘essential elements’ of a neural circuit, instead building large-scale models of neural circuits, modelling neuron-for-neuron [1]–[4]. This approach brings into sharp focus a further problem: how should we wire up the models? After all, the more accurate the underlying circuitry, the more confident we will be in linking dynamics of neural models to experimentally-recordable neural activity and, ultimately, to potential functions of the modelled structure. Typical modelling fall-backs of fully, regularly, or randomly connected networks are understandable choices when faced with this problem. Yet no neural circuit has these network topologies [5]–[8].

Establishing the detailed network of the striatum is a particular priority, given the large number of experimental and theoretical studies seeking to understand its computations [4], [9]–[16]. This large subcortical nucleus is the principal input structure of the basal ganglia, and is thought crucial for both motor control and learning [17], [18]. Profound deficits in both arise from diseases – such as Huntington's or Parkinson's – that directly affect the striatum or its primary afferents. Within the striatum lies a complex, predominantly GABAergic, microcircuit [19]. Medium spiny projection neurons (MSNs) are the only output neurons and comprise up to 97% of the cell population in rat, with GABAergic and cholinergic interneurons forming most of the remaining cell population. Despite their comparatively small number, the GABAergic fast-spiking interneurons (FSIs) in particular exert a very strong influence on the MSNs [20]–[22], receive input from similar sources, and are interconnected by both chemical synapses and gap junctions. However, the striatum's lack of layers and intermingling of neuron types has made it difficult to establish a detailed picture of its intrinsic network, hindering progress towards understanding the computations performed on its widespread cortical inputs [23].

One compelling reason for choosing to model at one-to-one scale is to explore a key question that can not be approached any other way: are there natural scales for the size of striatal regions involved in computing input-output functions? Much thought has been devoted to this question. The “domain” theory of striatum [9], [24], [25] began with the basic assumption that the natural computational element of the striatum was the network of MSNs within the radius of one MSN's dendritic tree – a sphere of approximately 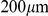 radius. Alexander and Crutcher [26] showed that microstimulation of primate sensorimotor striatum could elicit discrete movements of single joints, with each movement elicited from a small zone at most 1.2 mm in length. Graybiel and colleagues [27], [28] have argued that the pallidal-projecting regions of primate striatum are sub-divided into discernible cell clusters, each having a cross-sectional diameter of between 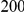 and 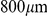. More recently, Carillo-Reid et al [29] have shown that global excitation of an *in vitro* slice of striatum can induce the appearance of three or four cell assemblies – co-active groups of cells – within an 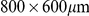 region. All these lines of evidence point to different sizes and different reasons for defining a ‘computational element’ within striatum. Hence, by building at such scales we can look for the natural size of the computational element.

First though we had to build a model of the striatal network. Complete reconstructions of neural circuits are technically challenging, so quantitative data on the inputs and outputs of a single neuron are often incomplete or absent, while many published values are rough estimates. One way around this problem is to use reconstructions of stained dendrites and axons as guides [30]. Recent approaches test for appositions between cells by passing three-dimensional reconstructions of the morphology of several axonal and dendritic fields through each other [31]–[33], yielding statistics on the probability and location of synapses between two neurons. However, sets of complete, three-dimensional reconstructions of both axonal and dendritic morphologies are not available for most neural structures. Furthermore, building a network based on intersections of a sample of reconstructions may unknowingly limit the possible topologies.

To overcome these problems, we developed a stochastic approach based on the density of overlapping neurites, determining the densities from prototype dendrite and axon models. We applied this approach to reconstructing the three-dimensional GABAergic microcircuit of the adult rat striatum. Building prototype dendrite and axon models for MSNs and FSIs allowed us to determine any omissions or inconsistencies in existing quantitative data, and to establish constraints on the dendritic locations of afferent input. Using these models to reconstruct the three-dimensional network, we could address key questions about striatal micro-anatomy: how sparsely is the striatum connected? What are the comparative numbers of contacts for each type of connection? Are there natural spatial scales of the sub-networks within it? And do these scales correspond to previous electrophysiological [26] and theoretical [25] indications of functionally separate sub-regions of striatum? Finally, we could use our anatomical model as the basis for a dynamic model that showed the functional consequences of the network's structure. Our network models provided unique insights into striatal circuitry, overcoming the unintuitive nature of connectivity in three dimensions.

## Materials and Methods

### The microcircuit and connection statistics

**Figure 1:**
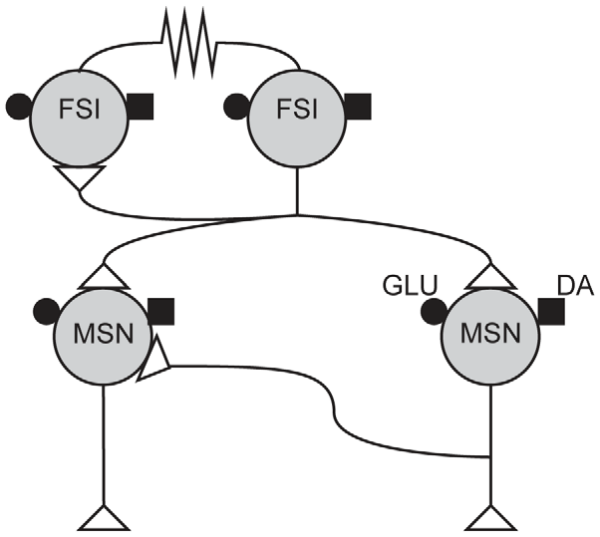
The striatal GABAergic microcircuit studied in this paper. Primary input to the striatum comes from glutamatergic (GLU: 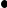) fibres originating in the neocortex, thalamus, hippocampal formation and amygdala, and dopaminergic (DA: ▪) fibres originating in the hindbrain dopaminergic neuron bands. The medium spiny neurons (MSNs) are interconnected via local collaterals of their axons projecting to other nuclei of the basal ganglia. The fast-spiking interneurons (FSIs) can form dendro-dendritic gap junctions between them; they may also be connected by standard axo-dendritic synapses. All these intra-striatal axo-dendritic connections (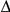) are GABAergic and hence inhibitory.

The striatal GABAergic microcircuit, shown in Figure 1, is formed by the connections between the GABAergic MSNs and FSIs. The MSNs are the only output neurons and comprise 90–97% of the neuron population in rat [19], [34], at a density of 84900 per 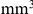 [35]. The FSIs form 1–5% of the striatal neuron population [19], [36]. Stereological counting suggests that parvalbumin-immunoreactive neurons, the likely histochemical marker for FSIs [37], make up 0.7% of the striatum [38], [39]. As we will see, our model supports this lower estimate: only an FSI density of at most 1% resulted in numbers of FSI connections that are consistent with current data.

Four connection types make up the microcircuit. First, MSNs extend local axon collaterals that synapse on other MSN dendrites. Long-established anatomically [40], considerable electrophysiological evidence for them now exists [11], [21], [22], [41], [42]. Second, axon collaterals from FSIs synapse onto MSN dendrites and somas [43], and have a strong inhibitory influence [20]–[22], [44]–[46]. Third, FSI dendro-dendritic gap junctions [39], [47] electrically couple the paired cells [14], [20]. (Gap junctions between MSN dendrites probably occur only in immediate post-natal tissue [11], [48] so we do not consider them here). Finally, the FSI axon collaterals synapse onto other FSI dendrites: previously, evidence for these connections was indirect [47], with others finding no electrophysiological evidence of synaptic connection [20]; however, a recent study using transgenic mice found synaptic connections between pairs of striatal FSIs in the majority of cases [21]. We study the implications of this newly-described connection here.

The connection statistics between MSN pairs are partially known. Conservative anatomical estimates place 600 synapses on one MSN from other MSNs [19], [49]. Stimulating an afferent MSN elicits a post-synaptic response consistent with it making an average of 3 synapses on the target MSN [44]. This gives a lower bound of 200 MSNs afferent to each MSN. Other estimates suggest a single MSN contacts around 12–18% of the other MSNs within a 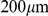 radius axonal field, based on the observed frequency of synaptically-coupled pairs in stimulation studies [44], [50]. This gives an upper bound of about 470 MSNs afferent to 1 MSN within a 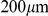 radius, in good agreement with previous estimates [44], [51]. Finally, Planert et al [22] recently reported synaptic-coupling between 20% of all tested MSN pairs with somas within 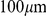 of each other.

There is less data on the statistics of FSI connectivity. Previous estimates of the number of FSIs afferent to a single MSN place bounds of 4–27 FSIs per MSN [19], [44]. Planert et al [22] recently reported synaptic-coupling between 74% of all tested FSI-MSN pairs with somas within 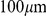 of each other. Fukuda [39] reported densities between 500 and 4000 gap junctions per 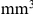 of striatal tissue, and observed typically 1–3 junctions per connected FSI pair. In Table 1, we use these data to calculate estimates for the number of FSIs connected to one FSI by gap junctions. These estimates show that we expect each FSI to be coupled to at most only a few others, and in many cases to have no gap junctions at all. As a consequence, and contrary to Fukuda's description of this network as “dense”, the FSI gap junction network seems to be very sparsely coupled.

**Table 1:** Estimates of the mean number of FSIs gap-junction coupled to each FSI, derived from Fukuda's [39] data.

		Number of gap junctions per coupled pair		
FSI density (%)	FSI density (# FSIs)	1	2	3
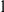	850	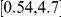	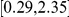	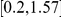
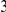	2547	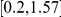	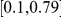	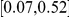
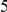	4245	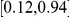	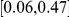	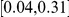

Fukuda reported densities of between 500–4000 gap junctions per 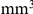 across the striatum; we used this to estimate lower and upper bounds for the number of FSIs gap-junction coupled to a FSI, for each FSI density and for between 1 and 3 gap junctions per coupled pair. For example, the first entry of the fourth column indicates that if we assume 2 gap junctions are made per coupled pair and the FSI density is 1%, then each FSI contacts between 0.29 and 2.35 other FSIs via gap junctions. We assumed here a MSN density of 84900 per 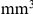 [35] when computing the density of FSIs.

### Outline of approach

Our aim was to construct a stochastic model of the three-dimensional network of the adult rat striatum, and study the statistics of contacts between the striatal GABAergic neurons. By “contact” we mean whether or not one neuron connects to another: a contact is one or more synapses or gap junctions. Our starting hypothesis was that, in a three-dimensional, non-laminar structure like the striatum, the *minimum* probability of contact between a pair of neurons is proportional to the density of their overlapping neurites. This is a passive process: numbers of contacts exceeding this minimum probability thus imply active processes, especially axon guidance towards specific types of target neurons. We encapsulate the role of active processes as an increase in the effective density of the axon. As we will see, this relatively simple model is able to capture the known statistics of the microcircuit's connectivity.

**Figure 2:**
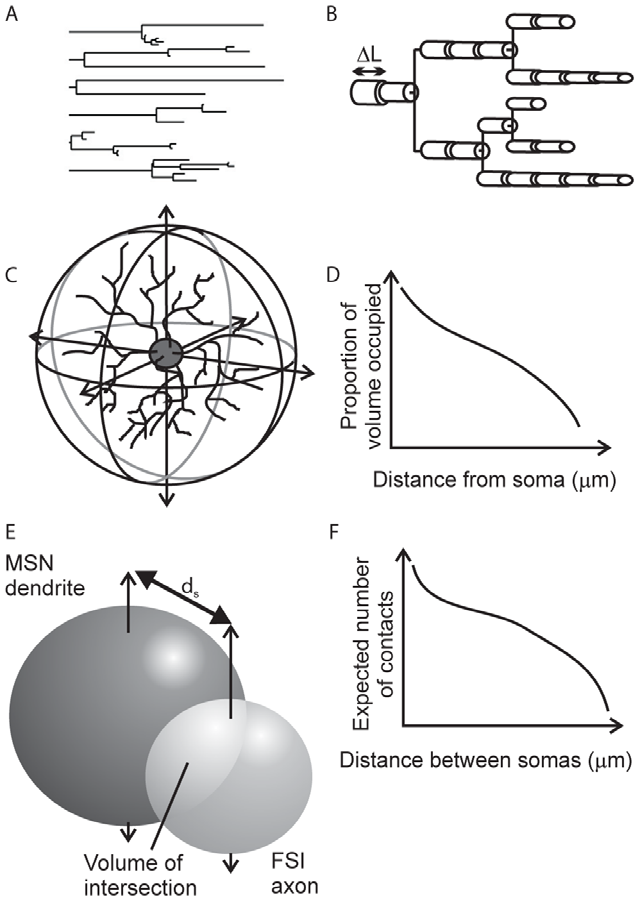
Anatomy model construction. Panels A–F show in order the steps involved in moving from a dendrite model to a probability function of contacts between two neuron types. **A** We create complete dendrograms using a stochastic algorithm, bounded by known properties of the dendrites. This example shows all six dendritic trees of the complete dendrogram for one MSN. **B** Each segment of each branch is modelled as a cylinder. The diameter of successive cylinders tapers with distance from the soma. Summing over all branches gives the total volume of dendrite (or axon) at each distance from the soma. **C** We then compute the proportion of spherical volume occupied by dendrite (or axon) at each distance from the soma. **D** Expected values for occupied volume are computed over many repetitions of the growth algorithm. The result is a continuous function of volume occupancy for each dendrite and axon type. **E** We find the intersecting volume between the dendrite and axon spherical fields for each distance 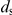 between somas. The volumes are discretised into 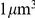 voxels. **F** For each voxel, given its distance from the respective somas, we compute the probability of intersection between neurites (dendrite-axon or dendrite-dendrite) from the volume occupancy functions (in panel D). We then sum over all probabilities to get the expected number of intersections between neuron pairs as a function of distance between their somas. We use the resulting functions to construct our networks.

Figure 2 illustrates the steps in our approach to reconstructing contact probability functions, starting from models of dendrites and axons. We began by generating the dendrites and axons of both MSNs and FSIs using stochastic models (Figure 2A). For the dendritic trees we used an existing algorithm [52] that has been successfully applied elsewhere. However, some key parameters for this algorithm require data that are typically unavailable for most neuron types. We overcame this problem by finding these parameters using an evolutionary algorithm search of a fitness space defined by known properties (e.g. number of branch points) of the neuron type's dendritic tree. For the axon we created our own model based on known properties of MSN and FSI axons. By creating models for the dendrite and axon structure, we had a full set of data on the dendritic branches and axons at each distance from the soma, including their approximate volume (Figure 2B). Hence we produced a large number of dendritic trees and axons to estimate expected neurite volume.

We could then compute the expected spherical volume that was occupied by dendrite (or axon) at a given distance from the neuron body (Figure 2C,D). Then, in turn, we computed the expected volume of overlap between the spherical fields given the distance between neuron bodies for each connection type (Figure 2E). For every 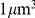 voxel in this overlapping volume, we computed the probability of its occupancy by both neurites (axon and dendrite or dendrite and dendrite, depending on the connection type) and thus the probability of intersection. Summing over all voxels in the overlapping volume thus gave us the expected number of intersections for each distance between neuron bodies (Figure 2F). We treat this as a probability of contact when constructing our three-dimensional networks. We elaborate on these steps below.

### Dendrite models: The Burke algorithm

We chose the Burke algorithm [52] for reconstructing model dendrites. The Burke algorithm constructs dendrites in short, cylindrical segments 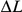 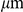 long, each successive segment tapering in diameter as the tree extends away from the soma. Details of the Burke algorithm are given in Text S1. At each step of the algorithm, the current segment can either extend, branch, or terminate. If the segment branches, it bifurcates into two daughter segments, both narrower than the parent, and one larger than the other. If the segment terminates, the branch is complete and the algorithm moves to the segment on the next unfinished branch. This algorithm is repeated from a single starting segment to obtain each of the dendritic trees necessary to form a complete dendrogram: we built 6 trees for a complete MSN dendrogram [40], [53], [54], and 5 trees for a FSI [37]. The dendrogram records the diameter, distance from soma, parent segment, and end type (branch, termination, or continuation) of each dendritic segment.

**Figure 3:**
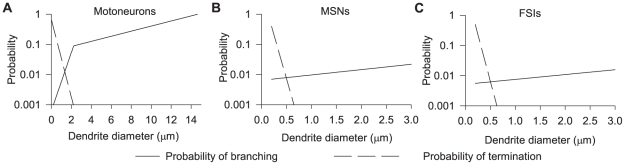
Probability of a branch or termination event as a function of the diameter of the dendrite. The dashed line plots the probability function for termination; the solid line plots the probability function for branching, given by equation (2). **A** The original functions from [52]. Branching probability is given by two exponential functions. **B** Probability functions found for the MSN dendrite models. **C** Probability functions found for the FSI dendrite models. The searches for both MSN and FSI dendrite models suggest that only a single exponential function is needed to describe the branching probability in these neurons.

The probability of a dendritic segment branching or terminating is a function of its diameter. To determine these probability functions, Burke et al [52] pooled morphological analyses of six spinal 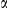-motor neurons to obtain a distribution of the number of branch and termination points at each dendritic diameter, and found the probability per-unit-length of either termination or branching; all their resulting probability functions had the exponential form
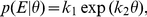
(1)for the probability of event 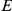 (termination 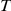 or branching 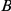), given dendrite diameter 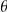 and the free parameters 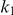 and 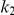. A single function 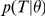 of this form was sufficient to fit the termination probability data; two functions of this form 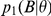 and 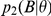 were required to fit the branch probability data. The single branching probability 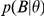 was obtained by evaluating both and using the minimum value:
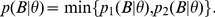
(2)
Figure 3A shows the termination and branch probability functions obtained from the 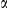-motor neuron data by [52] (for a segment length of 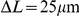).

As for most neuron types, detailed data on the diameters of dendrites at branch and termination points are not available for MSNs and FSIs, and so we could not define the probability functions and apply the Burke algorithm directly. Instead, we gathered morphological data on the known properties of their dendritic trees (Table S1 in Text S1): branch order, dendritic radius, number of terminals, and terminal diameter. We then searched to find the parameters for the probability functions that resulted in dendrograms fitting all the constraints of the gathered data.

#### Finding the parameters for the Burke algorithm probability functions

We used an evolutionary algorithm search to find the set of parameter values for the probability functions of the Burke algorithm, one set for MSN and one set for FSI dendrograms. Each candidate in the search was a vector comprising values for the 6 parameters of the probability functions, namely 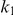 and 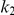 for each of 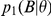, 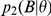 and 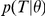. The complete form of the search is given in Text S1. In general, we began with an initial population of candidates, each with randomly chosen values. The values from the first candidate were then used in the Burke algorithm to generate multiple instances of the dendrogram. The fitness value of that candidate was taken as the proportion of resulting dendrograms that fell within all the bounds on morphological properties (Table S1 in Text S1). Each candidate was evaluated in turn, and ranked by fitness. The most-fit candidates were randomly paired, and the others discarded. Each pair mated to produce two offspring by crossing over the two vectors at a randomly chosen point. Each parameter in the retained (most-fit) candidates and their offspring was then tested for mutation to some other value, with low probability. The resulting new population then formed the basis for the next set of Burke algorithm tests. This cycle of ‘population testing then pair-mate-mutate’ was repeated until the most-fit candidate reliably generated dendrograms that fell within all the bounds on morphological properties (Table S1 in Text S1), or the maximum number of population generations was reached. We used the most-fit candidate to generate our dendrograms that then underpinned the volume and intersection calculations.

### Axon models

We use a simpler model for the axons, partly due to the absence of equivalent data to constrain a growth algorithm, partly because their structure is simpler, and partly because we can later use the axon model to encapsulate the process of attraction between axon and dendrites. The only quantitative description of local MSN axon collateralisation we are aware of is due to Preston et al [53], who described axons maintaining a diameter of 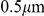 over their initial length, then branching into 4 collaterals within 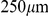 of the soma, each with a diameter of 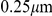 (which terminate in extensive branching). This suggests an approximately two-fold increase in total diameter after all the branching had occurred. Similar branching patterns have been reported in [55]. We are not aware of any equivalent data for striatal FSI axons, and so use the same axon model for both as their axonal fields are similar [20].

**Figure 4:**
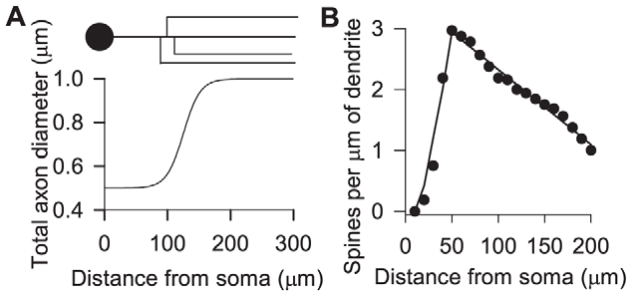
Elements of neurite modelling. **A** The axon diameter model. The schematic MSN axon (top) shows the assumption that all branches occur over a short interval of 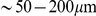. This is modeled as a continuous increase in axon diameter for convenience. **B** Spine density data (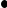) from [59] and our piece-wise linear fit (see equation 6).

Based on these observations, we proposed a sigmoidal model of the changes in axon diameter, in which the total axon diameter 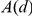 at distance 
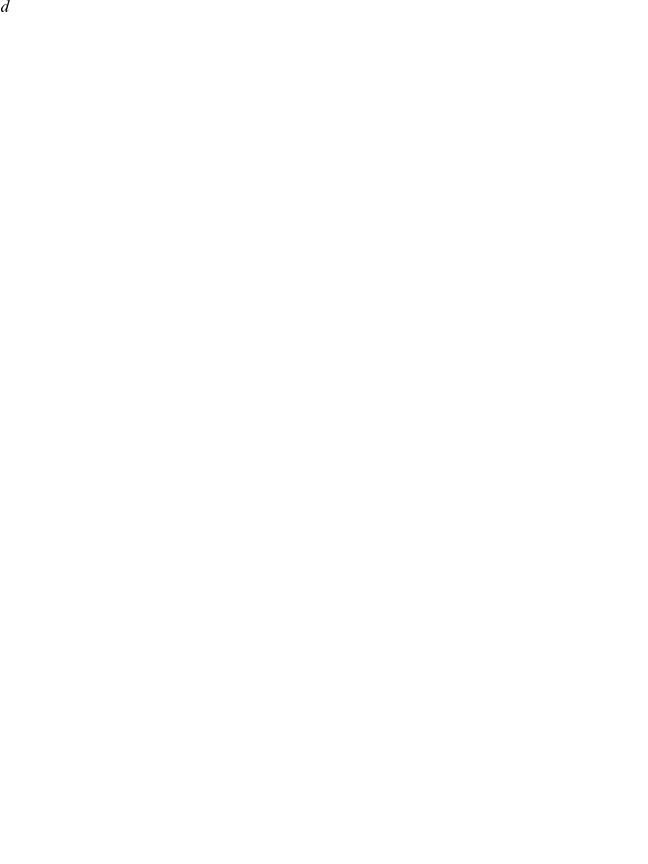
 from the soma is given by
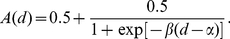
(3)We used 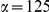 and 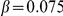 throughout for both MSNs and FSIs. With these values, the model captures all axonal branching occurring between 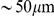 and 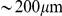 from the soma [53], [55], as illustrated in Figure 4A. We used a maximum distance from the soma of 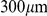 for both the FSIs [20], [37], [56] and the MSNs [53], [55].

### Embedding in space: estimating the volume occupied by neurite

#### Dendrites

The Burke algorithm parameters from the evolutionary algorithm searches were used to generate 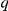 dendrograms of FSIs and MSNs. A dendrogram was rejected if its morphological properties did not fall within the bounds for all of branch order, dendritic radius, number of terminals, and terminal diameter (Table S1 in Text S1), ensuring that all 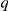 retained dendrograms were accurate within the constraints of the available data.

Our dendrogram models describe the bifurcation and termination of dendrites along the radial axis stretching away from the soma; however, real dendrites wander extensively around their straight-line axis. The extent of this ‘tortuosity’ is measured as the ratio of the actual length of a given dendritic segment to the measured straight-line length. We adjusted the lengths of the dendrite segments to account for tortuosity by factors of 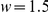 for MSNs [57] and of 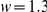 for FSIs (data from cortical FSIs [58]).

For each dendrogram we found the total volume occupied by the dendritic shafts between distances 
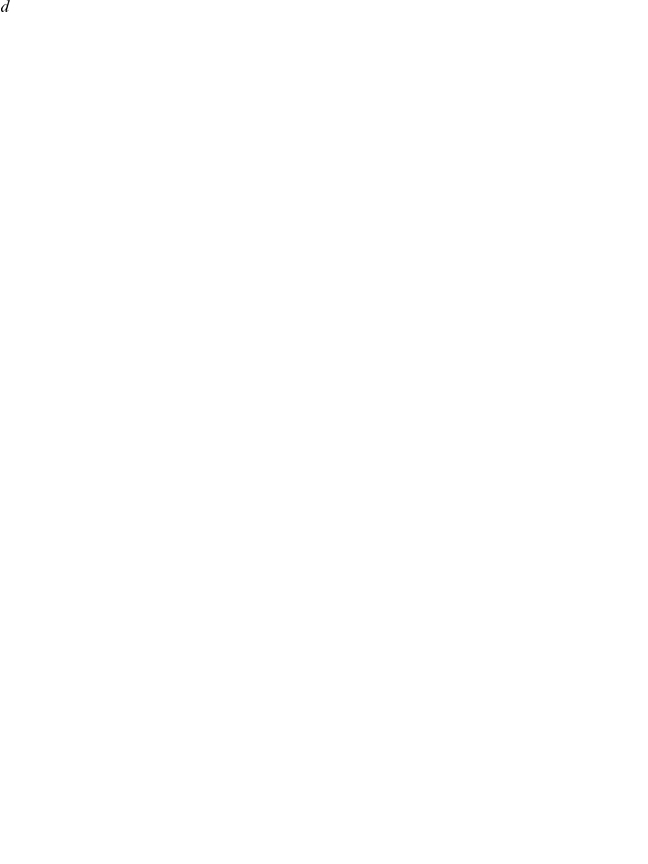
 and 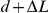 from the soma; we abuse the terminology slightly and refer to this as the volume 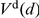 *at* distance 
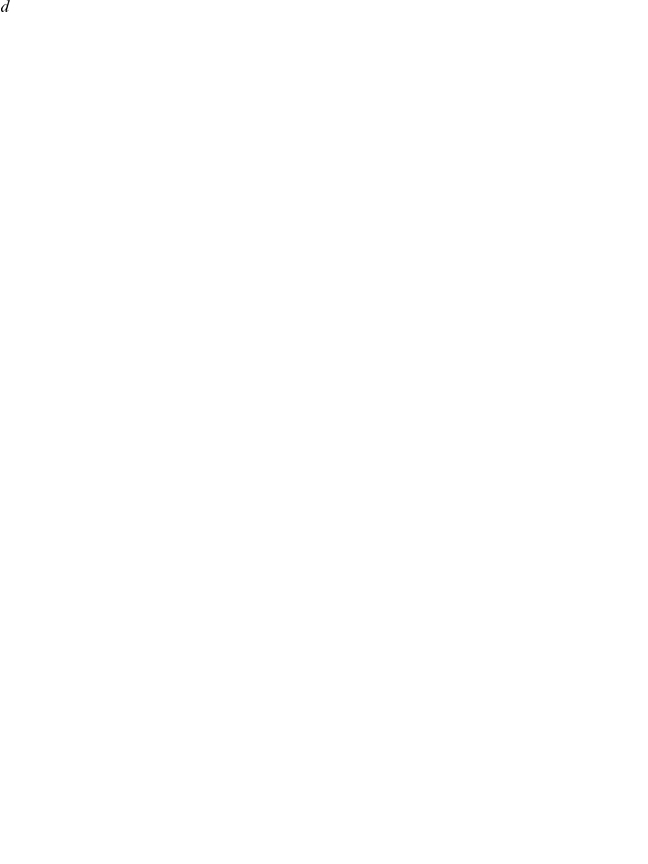
 from the soma. Assuming that each dendritic segment of length 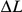 is a cylinder, the total dendritic shaft volume 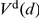 at distance 
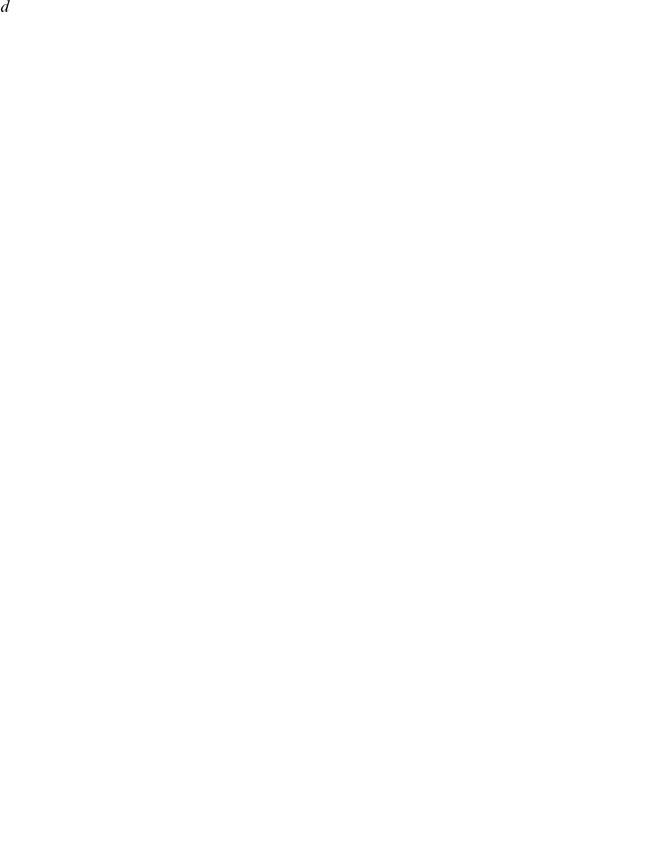
 from the soma is
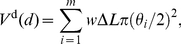
(4)where 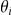 is the diameter of the 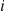
th segment, and the sum is taken over all 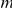 segments at distance 
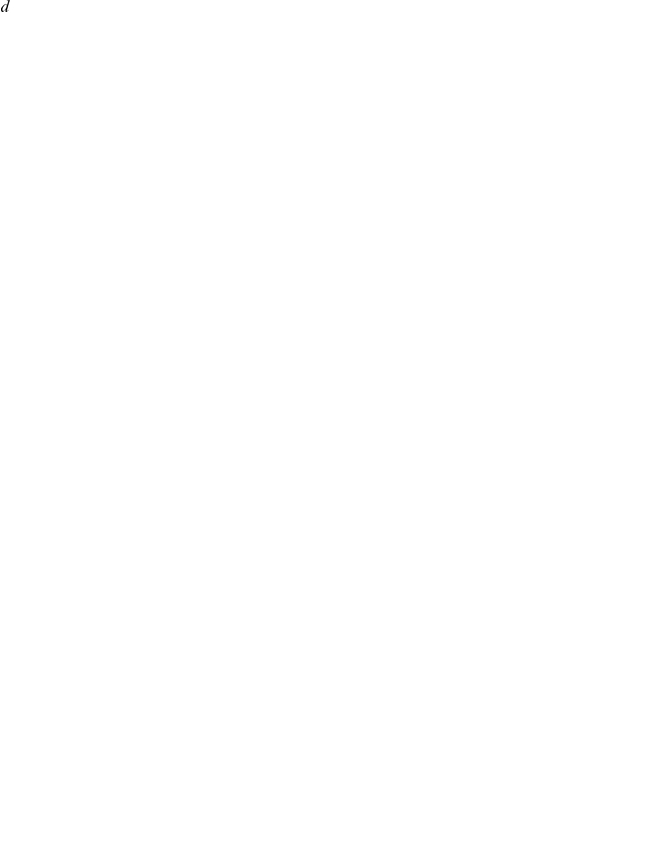
. Equation (4) is calculated for each 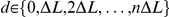, up to the final 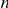
th segment in the dendrogram.

The average over all 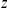 contributing dendrograms at each distance is then
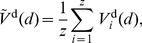
(5)where 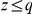: at long distances not all constructed dendrograms will have any dendrite.

To accurately estimate the volume of the MSN dendrites, we needed to account for the additional volume provided by their dendritic spines. Figure 4B plots the mean number of spines per 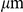 as a function of distance 
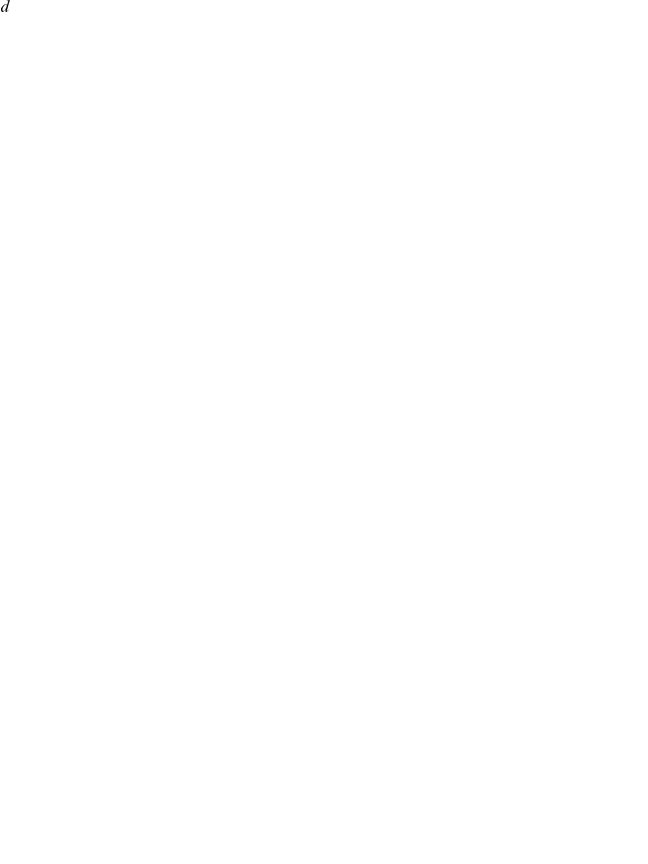
 from the soma, obtained from a previous detailed study of MSN spine morphology [59] (data from C. Wilson, personal communication). Figure 4B also shows our piecewise linear fit 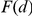 to this data, given by
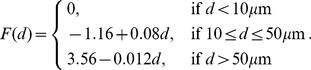
(6)The parameters for the linear fit were found by least squares regression using the MATLAB (Mathworks, Natwick CA) routine lsqcurvefit.

For each MSN dendrogram, we used equation (6) to find the total number of spines at distance 
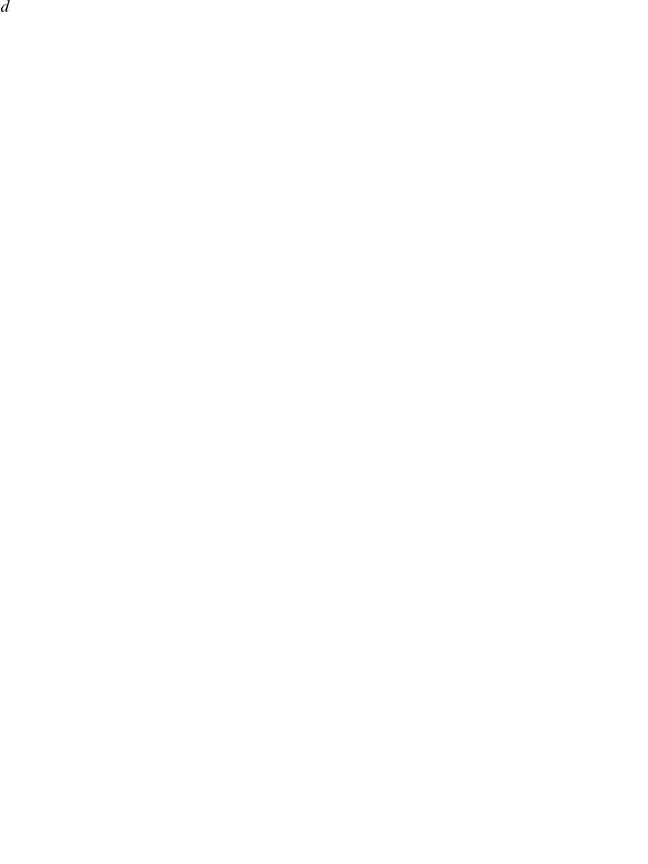
 from the soma,
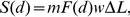
(7)where 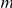 is again the number of segments at distance 
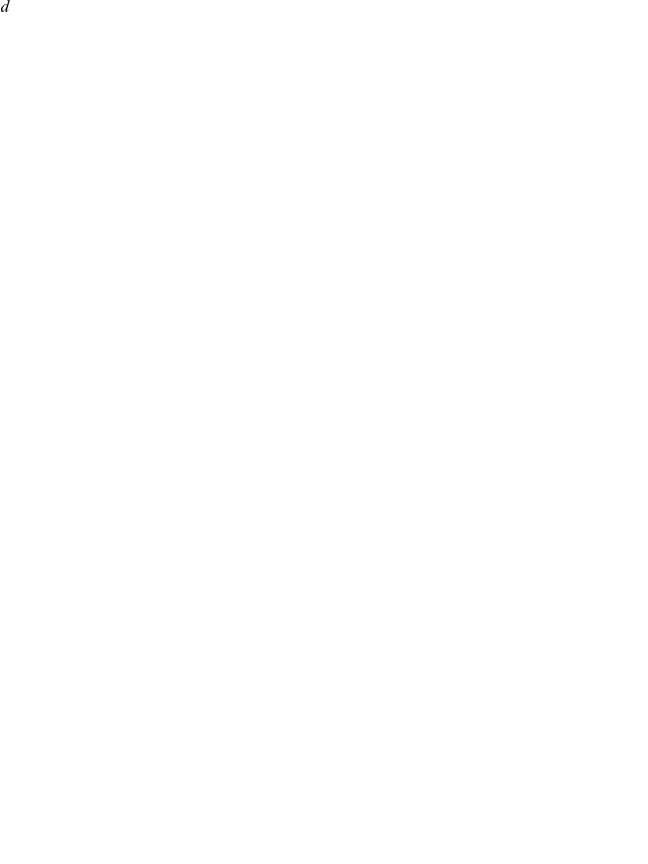
, and equation (7) is again calculated for each 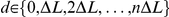. The average over all 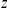 contributing dendrograms at each distance is then
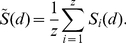
(8)The total spine volume 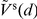 at distance 
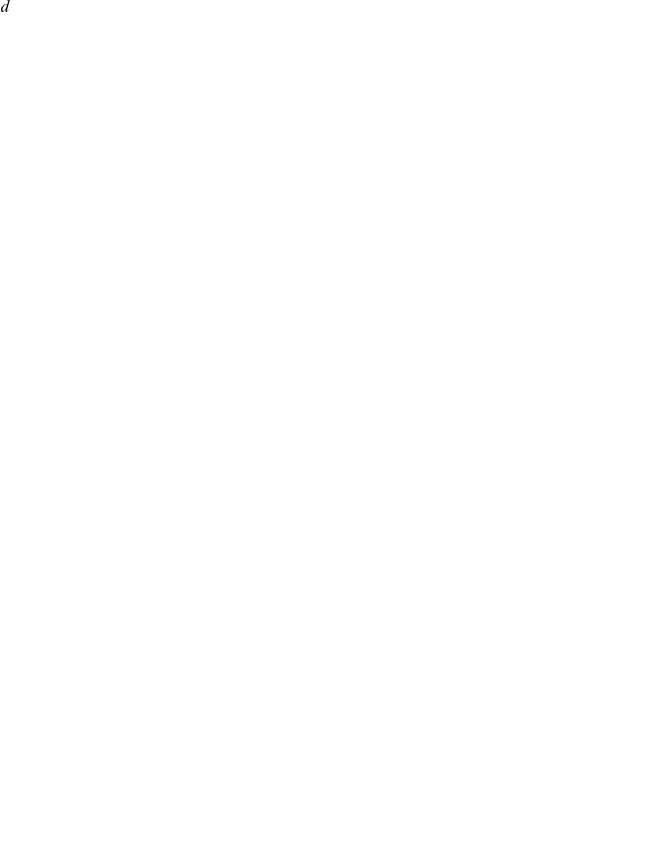
 from the soma is thus given by
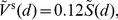
(9)where we make use of the recorded mean volume of 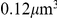 for an individual spine [59].

Putting this all together, the estimated total dendritic volume 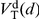 per 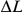 step from the soma for FSIs was just 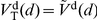, given by equation (5); for MSNs it was 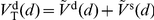, the sum of dendritic shaft and dendritic spine contributions. We fitted continuous functions 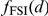 and 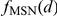 to these estimates, allowing us to determine the volume at any arbitrary distance 
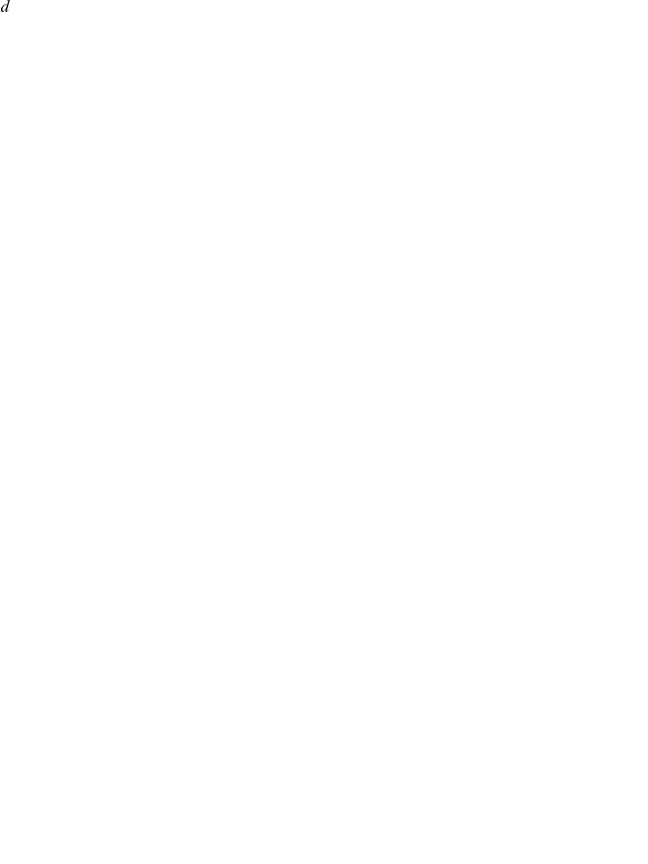
. The specific fits we found are given in the Results.

#### Axons and attraction to dendrites

We already have a functional description of total axon diameter as a function of distance 
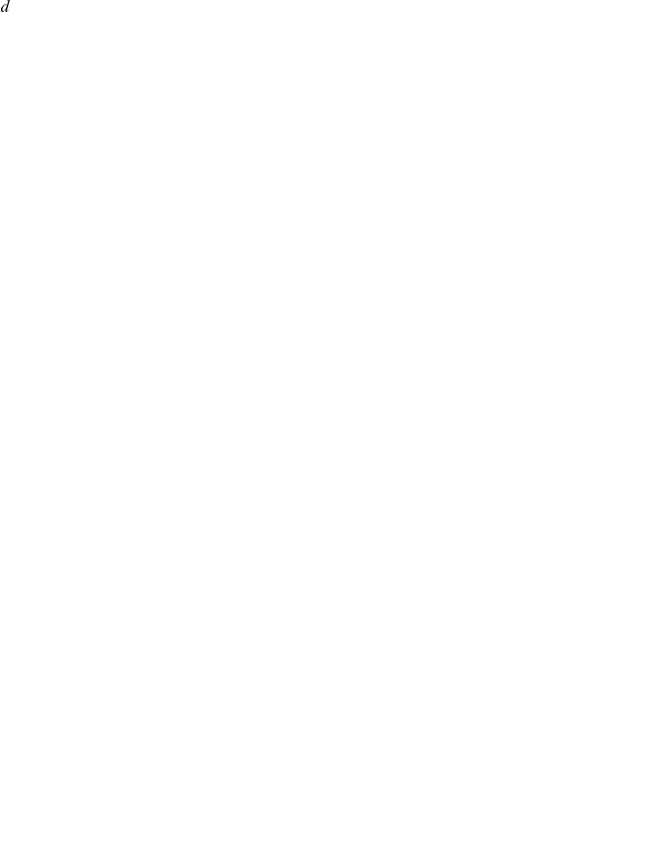
, given by equation (3). Using this, a naive model of the total volume at 
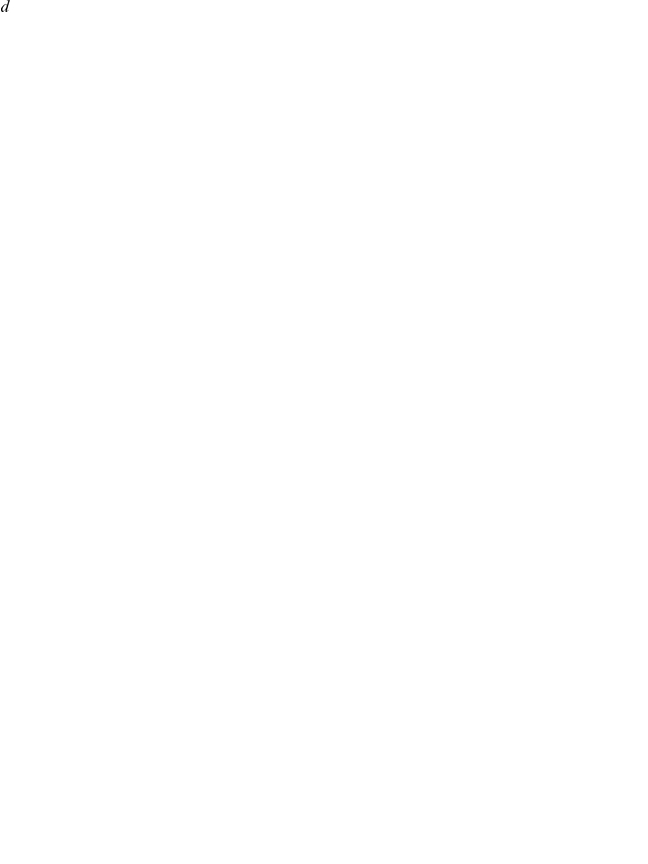
 would be
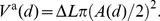
(10)For consistency with the dendrite calculations, the axonal volume is found using the same segment length 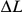, and again assuming each axonal segment is a cylinder.

The axon model (equation 10) gives the straight-line change in axon diameter. Yet, both MSN and FSI axon collaterals wander extensively within their overall field [37], [53], [56], reflecting that the axon trajectory is dependent on active processes (such as chemodensity gradients or relative neuron activity) guiding it towards particular dendrites during development (e.g. [60]–[62]). We therefore introduce a density constant 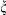 to scale the total axon volume,
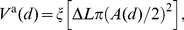
(11)capturing the *effective* volume of the axon. Exact values for 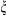 are unknown, so we establish plausible values using recent data on the probability of connections between MSN-MSN and FSI-MSN pairs up to 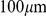 apart (see *Finding the axon-density constant* in the Results). A further check on their plausibility is that the resulting estimates of numbers of connections per neuron for the whole model network should match existing experimental estimates.

### Probability of intersection

Both MSNs [40], [63] and FSIs [20], [37] have approximately spherical dendritic and axonal fields. Following a mean-field approach, we thus made the simplifying assumption that the probability of finding the neurite is the same in all directions for a given distance away from the soma. We could then compute the following from the estimates of dendrite and axon volumes: the probability of finding dendrite (or axon) at a given distance from the soma, in a given volume of space; and hence the probability of intersection between two neurons' neurites in the same volume of space. To compute probabilities it was necessary to define the minimum volume required for a single intersection. The total volume of space was thus discretised into cubes or *voxels* that were 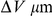 on the side. We set 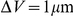 to be consistent with the rat striatum's synaptic density of approximately 1 per 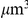 [64]; this scale of individual intersections is also common to studies of rat cortical connectivity [31], [33].

As we are assuming that the probability of finding a neurite is invariant for a given distance from the soma, we proceed by considering successive spherical shells of width 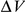, the first shell wrapped around a sphere describing the soma. The voxels in a given shell will have the same probability of containing a neurite. The total volume of a shell at distance 
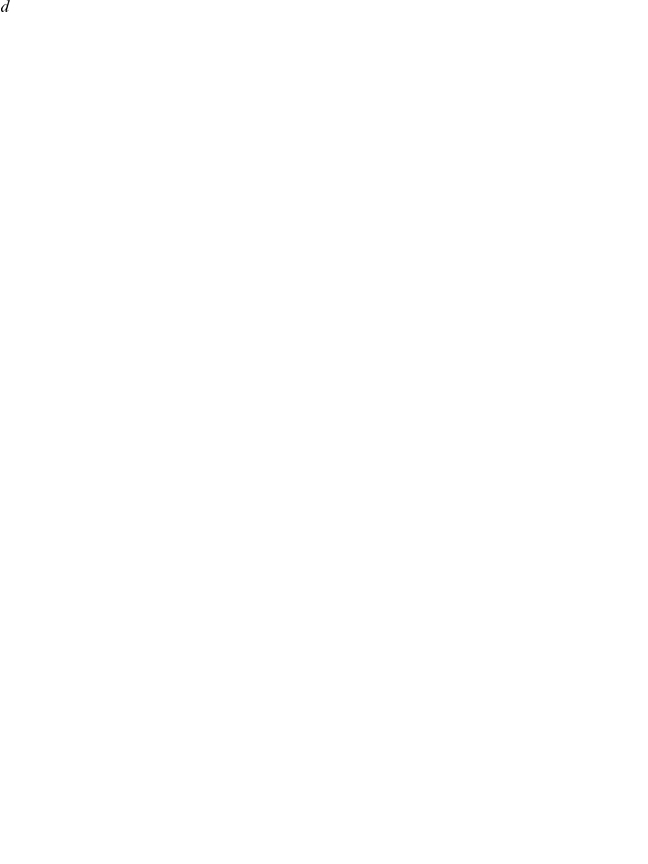
 from the soma is
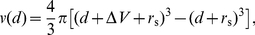
(12)where 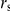 is the radius of the soma: we used 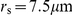 for both MSNs [65] and FSIs [20].

If the number of 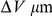-on-the-side voxels in a shell at distance 
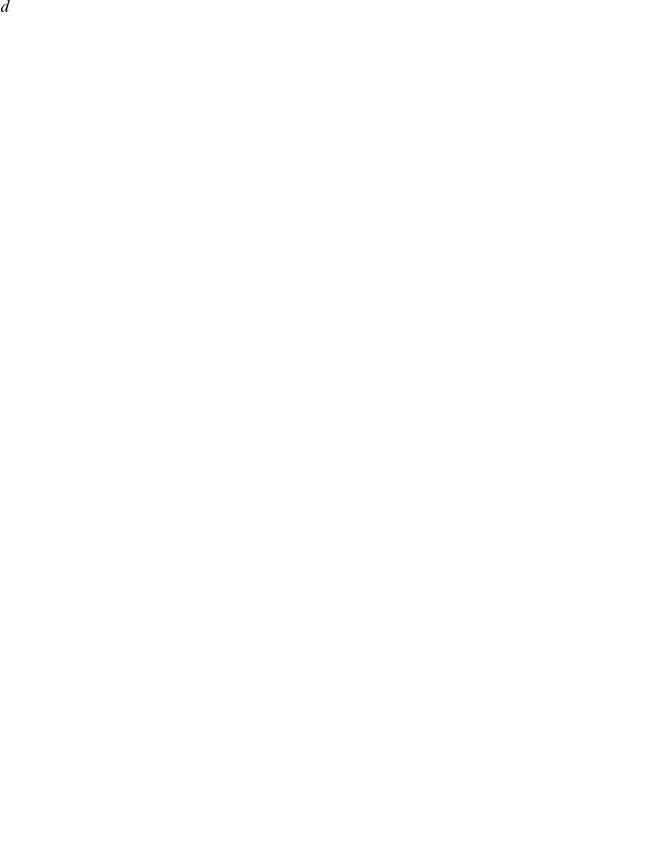
 from the soma is
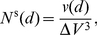
(13)and the number of voxels occupied by dendrite in that shell is
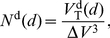
(14)(where 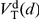 is the total dendritic volume at that distance from the soma) then the ratio 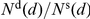 gives the probability of finding a dendrite-occupied voxel in that shell
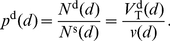
(15)Similarly, for axons occupying 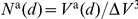 voxels of the shell, the probability of finding an axon-occupied voxel in that shell is
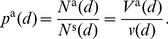
(16)(For arbitrary distances 
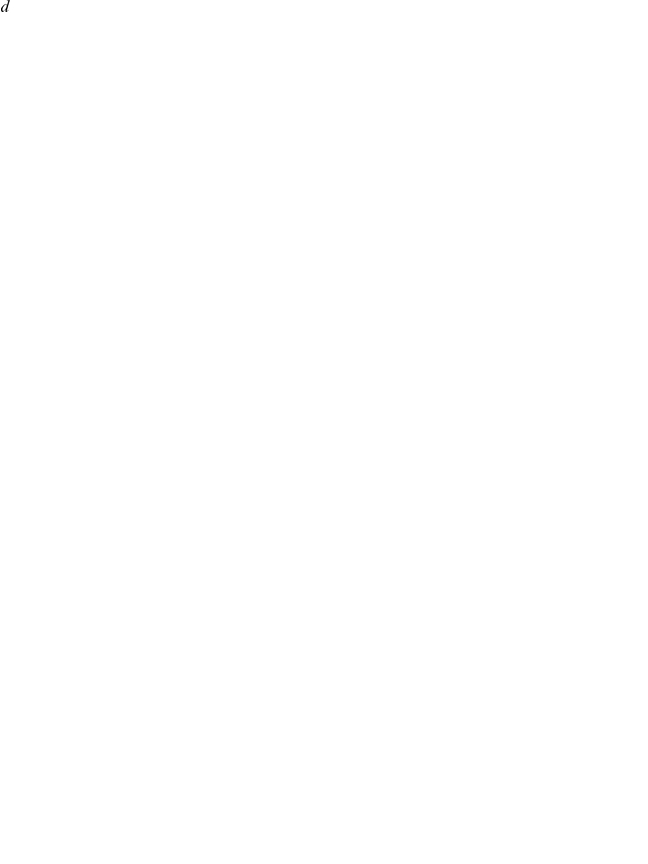
 from the soma, we could compute dendrite equations (14) and (15) using the continuous function fits 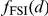 or 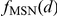 to the corresponding 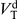 for FSIs and MSNs, respectively. Probabilities for the axons could be computed at arbitrary distances 
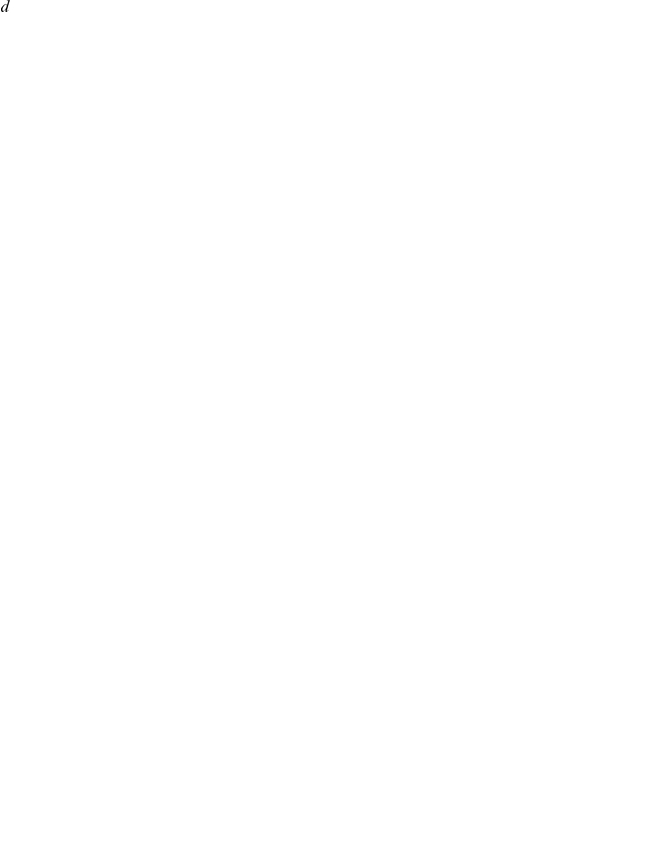
 directly from equation (16) because the axon volume function (equation 11) is continuous).

Having obtained an estimate of the probability that a voxel contains an axon or dendrite, we could calculate the probability that a voxel contains an intersection between the neurites of two neurons. Let us denote the distance between the somas of the two neurons as 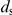. A given distance defines a volume of intersection between the two neurite-occupied spheres (Figure 2E). The centre of a given voxel in this intersecting volume is at distance 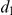 from neuron 1 and distance 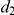 from neuron 2. The probability of this voxel containing a neurite of the required type from both neurons is then
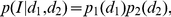
(17)given the probabilities of finding a neurite from neuron 1 (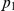) and neuron 2 (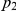) in that voxel, from equation (15) or equation (16).

The total expected number of neurite intersections between two neurons at a given distance 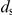 apart is then
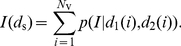
(18)where 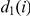 is the distance of the soma of neuron 1 from the 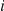
th voxel, 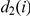 is similarly defined for neuron 2, and 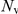 is the total number of voxels in the intersecting volume of the two neurite spheres. We calculated equation (18) for a range of inter-somatic distances 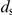, and fitted the resulting range of 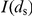 values with a continuous function 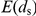 so that we could obtain the expected number of intersections between a pair of neurons for an arbitrary distance between their somas. We did this for each of the four types of connection in the microcircuit (Figure 1), and the fitted functions 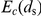 are given in the Results – we add the additional subscript 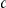 to denote which of the four connection types is being described.

### Building a network

We first define a volume of striatum we went want to model. The striatum contains 84900 MSNs per 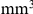 [35]; we added either 1% [19], 3% or 5% [36] of those as FSIs. We randomly assigned three-dimensional positions to each neuron, with a minimum distance of 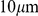 between neurons enforced, to model the non-laminar structure and intermingling of neuron types. To wire up the network, we treated the continuous functions 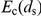 giving the expected number of intersections between a pair of neurons as the probability of a contact between the pair of neurons. Hence, 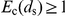 was treated as a contact with a probability of unity. Thus, given a particular distribution of neurons in space, with each pair at some distance 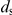, for each connection type 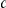 we used 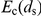 as the binomial probability of a contact.

### Dynamics on the network model

We explored the dynamical implications of some of our anatomical findings, using a computational model of the striatum drawn from our previous work [4]. In the model used here, the model neurons were wired together using our found intersection functions and the resulting network models; otherwise, the model neurons, synapses, gap junctions and inputs were as specified in [4]. Briefly, the neurons were simulated using the canonical, two-dimensional spiking model of Izhikevich [66], adapted to match the input/output properties of striatal MSNs and FSIs. We used conductance-based, single exponential synaptic models for intra-striatal connections (GABAa) and cortical input (AMPA and NMDA). As in the real striatum, we made synapses between model MSNs relatively weak, and the FSI synapses on MSNs relatively strong: following existing data [22], [44], the FSI-MSN synaptic conductance was five times greater than the MSN-MSN synaptic conductance. Gap junctions were modelled as a passive compartment between the coupled neurons, with a time-constant and conductance previously obtained by tuning to data on electrically-coupled cortical FSIs [4]. Cortical input was specified as the mean number of events/s arriving at excitatory synapses.

We ran two sets of simulations: one set used networks constructed within 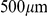-on-the-side cubes of model striatum; the other used a network within a 1 mm-on-the-side cube. For the 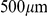 scale networks, we looked at the spontaneous activity of the striatal network in response to 10 seconds of background input of 475 events/s to every neuron (corresponding to around 1.9 spikes/s for 250 active afferents). For the 1mm-scale network, we selected the MSN closest to the centre of the cube as our reference neuron. We then stimulated all neurons in a series of 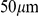 wide spherical shells extending away from this central MSN. For each simulation, the central MSN and all neurons (MSNs and FSIs) in a shell were driven for 4 seconds with a mean of 1250 events/s (corresponding to around 5 spikes/s for 250 active afferents).

## Results

### The MSN and FSI dendrite models

The evolutionary algorithm searches successfully found usable Burke algorithm parameters for both MSN and FSI dendrograms. The resulting parameters are given in Table 2. For MSNs, the top parameter set had a fitness of 83.3%, and was found on generation 44. For FSIs, the top parameter set had a fitness of 100%, and was found on generation 114. Both top sets were thus found well before the termination of search, and are likely to be close to the best available given the initial population. (Note that a fitness of 100% does not mean that the parameter set guarantees an accurate dendrogram every time, due to the stochastic nature of the Burke algorithm). We used these parameters to generate 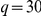 MSN and FSI dendrograms.

**Table 2:** Search results: final parameters for the MSN and FSI branch and termination probabilities, rounding to two significant figures.

	MSN		FSI	
	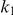	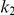	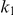	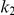
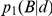	0.059	18	0.039	91
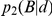	0.0065	0.41	0.0052	0.37
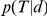	5.7	−13	8.6	−14

The resulting probability functions for branching and termination of the MSN and FSI dendrites are shown in Figure 3. The search results predict that, because 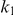 for the second branching probability function 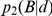 is very small (Table 2), only a single exponential is effectively needed to describe the branching probabilities of both neuron species, rather than the two exponentials fitted by Burke et al [52] to their motorneuron data. This suggests some fundamental difference in the morphology of MSNs and FSIs, compared to the morphology of the motorneurons studied in [52].

**Figure 5:**
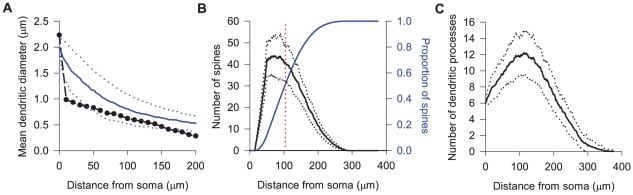
Predictions of the MSN dendrite models. **A** The diameters of MSN dendrites as a function of the distance from the soma (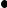; data supplied by C. Wilson), and the mean diameters predicted by the dendrite model with the parameters from the evolutionary algorithm search. The data mostly fall within one standard deviation of the model's mean values. (The diameters were averaged over all instantiated dendrograms; the dashed lines indicate 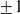 s.d. from the mean). **B** Predicted distribution of spines across the dendrites of one MSN (histogram of the mean number of spines per 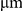 step away from the soma in black, with 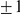 s.d. plotted as dotted lines; cumulative distribution of spines given by the blue solid line). The dendrite models predict that the mean total number of spines per MSN is 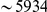, with half occurring within 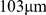 of the soma (indicated by the horizontal red dotted line). **C** The mean number of dendritic processes for an MSN per 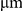 step away from the soma (solid line; dotted lines plot 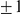 s.d.); given the known relationship between spine density and distance from the soma (Figure 4B, equation 6), the fall of the number of processes able to support spines dictates the spine distribution shown in panel B.

The resulting MSN dendrogram models made some interesting predictions. Figure 5A shows that the predicted dendritic taper of the MSN model closely approximated the dendritic taper data recorded from real MSNs ([67]; data from C. Wilson, personal communication). The data from the real MSNs suggests a sharp initial decrease in diameter as the dendrite leaves the soma that is not captured by the model, but otherwise the tapering is of a similar form.

Second, the model MSN dendrograms predicted that existing data on total dendrite length and estimates of spine counts are mutually inconsistent. The median total dendrite length, averaged over all instantiated dendrograms, was 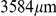 (range 2693–4925), exceeding the previously obtained median value (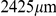) and range of 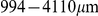 reported by Meredith et al [54] across 22 MSN reconstructions. The predicted number of spines on the whole dendritic tree was 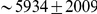 (mean 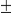 2 s.d.), a mean value lower than the bottom end of the previously predicted range of 6250–15000 spines per neuron based on the same original spine data [68]. The dendrogram model has thus shown that, even if the total dendritic length extends beyond the reported data, we cannot recover these total spine estimates.

A third prediction is that the spines are in abundance in the proximal dendrites. We plot the histogram of the MSN dendrograms' mean spine counts in Figure 5B and see that it is skewed, with half of all spines occurring within 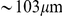 of the soma. The MSN model also shows us that the long-tailed fall-off of the number of spines when moving further away from the soma is primarily due to a corresponding fall in the number of processes across the whole dendrite (Figure 5C).

### Dendrite and axon volumes, and probabilities of finding neurites

**Figure 6:**
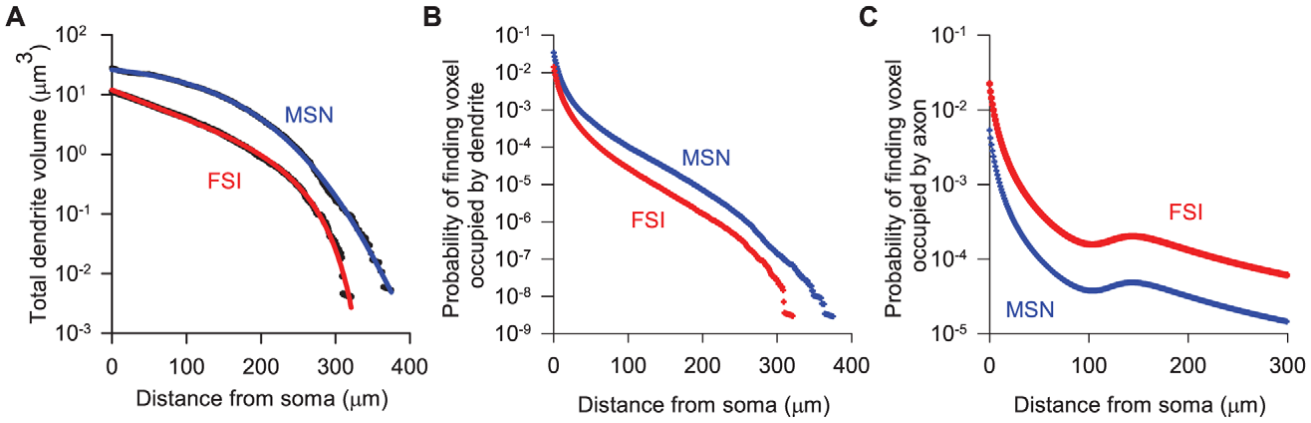
Model predictions for the changes in neurite density and detection probability with distance from the soma. **A** Model predictions for the total volume of dendrite at a given distance from the soma. The solid lines give the best-fit functions of the form in equation (19). Both this rational function form and the 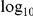-transform of the data were necessary to accurately fit the tails of the distributions. **B** Probabilities for MSN and FSI dendrites, computed directly from the mean total dendrite volume estimates. **C** Probability for finding an axon-occupied voxel, as given directly by evaluation of equation (16) (shown for the chosen axon-density constant values of 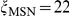 and 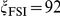).

We used the instantiated dendrograms to find the mean total volumes 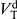 of the MSN and FSI dendrites per 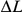 step (equations 4–9). Having found these mean total volumes over a range of distances 
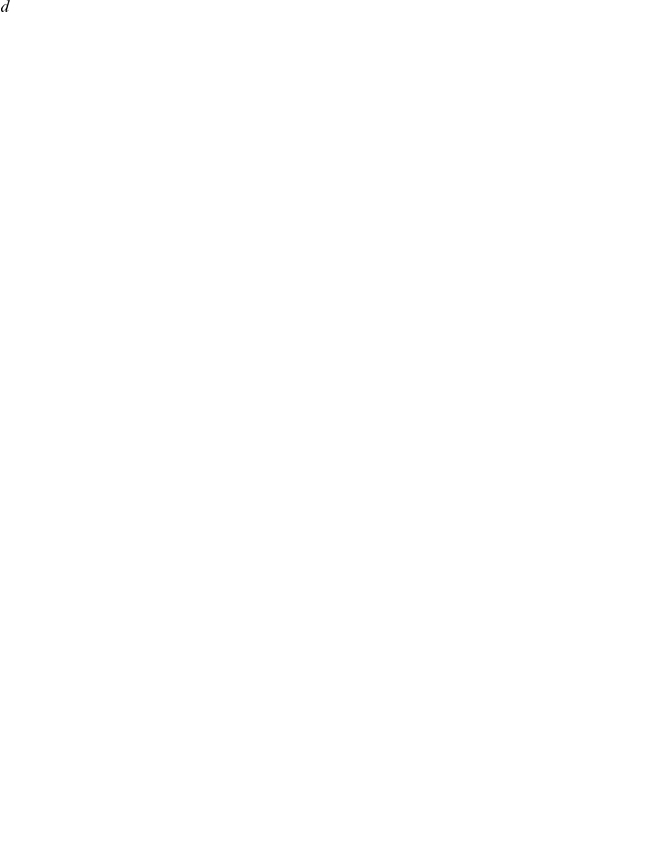
 from the soma, they were fitted with functions of the form
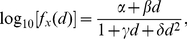
(19)to obtain functions 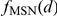 and 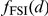 giving us the volume of MSN and FSI dendrite, respectively, at arbitrary distance 
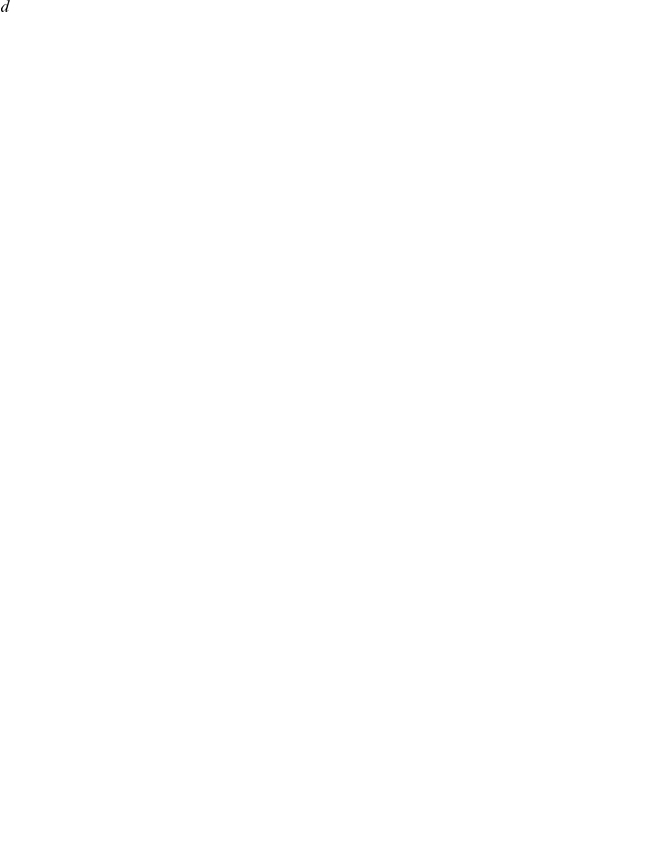
 from the soma. Table 3 gives the best-fit parameter values (found using non-linear least squares, as implemented by MATLAB function lsqcurvefit). Both the functional form and the 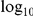 transform in equation (19) were necessary to accurately fit the tails of the total volume distribution (Figure 6A). The transform overcomes the problem that using summed-squared error favours close fits to higher magnitude data-points, as the majority of ‘error’ occurs for them.

**Table 3:** Parameters for best-fit functions to the model predictions of total dendrite volume.

Neuron	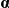	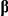	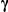	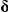
MSN	1.416	−0.0056	−0.0031	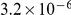
FSI	1.077	−0.0055	−0.00032	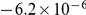

The importance of close-fitting to the tails becomes clear when we consider the probabilities of finding a neurite-occupied voxel, and the subsequent intersection calculations. When we compute the probability of finding a dendrite-occupied voxel (Figure 6B), we see that it falls faster than the dendrite volume (compare Figure 6A): the volume of the embedding spherical shell increases cubically with each 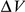 step. Yet when we turn to compute the number of intersections, the number of voxels also increases cubically with each 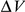 step. Hence, at intermediate distances from the soma, the very small probabilities of finding neurites are counteracted by the very large number of voxels checked for intersections. Poor fits to the tail thus incur noticeable changes in the number of expected intersections.

### Contact probabilities within the microcircuit

Finally we turn to actually computing the expected number of intersections for each of the MSN and FSI connection types in the striatal GABAergic microcircuit (Figure 1): local axon collaterals connecting MSNs [40]; projections from FSIs onto MSNs [20]; axo-dendritic synapses between FSIs [21]; and dendro-dendritic gap junctions between FSIs [20], [39], [47].

#### Finding the axon density constant

We performed a simple procedure to find plausible values of the axon density constant for MSN (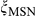) and FSI (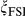) axons, based on data from [22] for probabilities of contact between MSN-MSN and FSI-MSN pairs within 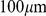 of each other. We placed MSNs in a 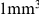 cubic regular lattice. Putting 44 MSNs on a side gives a density of 85184 MSNs per 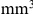, as close to the experimentally measured density of 84900 per 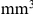 [35] as we can get using a regular lattice. For a range of values of 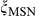 in the axon volume model (equation 11), we found the expected number of intersections (equation 18) between the dendrites of the centre MSN and the axons of all MSNs with a soma within 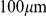 of the centre (using our found dendrite volume function 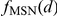 from equation 19). We also repeated the procedure for a range of values for 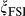, except that the centre neuron was now a FSI (so using our found dendrite volume function 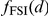 from equation 19).

We found that the probability of contacting the central neuron was an exponentially saturating function of the axon-density constant: 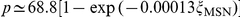 for MSN-MSN connections, and 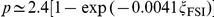 for FSI-MSN connections. Planert et al [22] gave approximate probabilities of 0.2 for a MSN-MSN pair and of 0.75 for a FSI-MSN pair connecting within 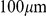 of each other. To match these probabilities, the functions we found predict values of 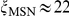 and 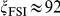.

#### Intersections between each type of neuron pair

**Figure 7:**
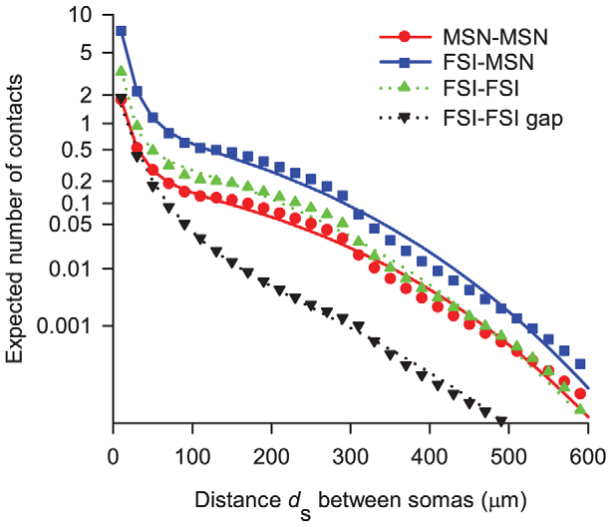
Expected number of intersections occurring as a function of the distance between the somas of two neurons. Symbols give the numerically determined predictions of the dendrite and axon models. Lines give the best-fit functions of the form in equation (20), for use in constructing networks.

Given the axon-density constants, we computed the expected number of intersections (equation 18) for each of the four connection types over a wide range of distances between the somas (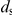 incremented in 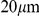 steps within the interval 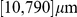; the upper limit of 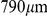 was used as this was the approximate inter-soma distance at which the largest recorded model dendritic and axonal fields would touch). Figure 7 shows that for all connection types the expected number of intersections, and hence the number of contacts, falls quickly with increasing distance between the somas. At 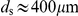 apart, a source neuron is expected to make less than 0.05 contacts with a target neuron. Local axon collateral contacts between MSN-MSN and FSI-FSI pairs are predicted to have approximately the same distribution as a function of distance between the pair, particularly further apart than 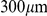. Considerably fewer gap junction contacts between FSI pairs are predicted as a function of distance; we show below the clear effect this has on network topology.

We found that the distributions for the expected number of intersections were well-fit by functions of the form
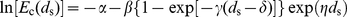
(20)for each of the four connection types. The parameter values for the best-fits to each connection type 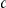 are given in Table 4. Both this functional form and logarithmic scaling of data were again necessary for accurate fits to the tails of the distribution. In this case, accurate fitting was essential for building networks. Though the expected number of intersections falls rapidly, the number of cells that are potentially contactable increases cubically with increasing distance from the source neuron. Thus, though probability of contact is small, the large number of repeated tests means some contacts are made.

**Table 4:** Parameters for the expected number of intersections between neuron pairs.

Connection type	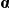	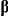	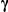	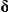	η
MSN-MSN	0.511	1.033	0.042	26.8	0.0039
FSI-MSN	−0.921	1.033	0.042	26.8	0.0039
FSI-FSI	−0.695	1.38	0.057	15.6	0.0036
FSI gap	1.322	2.4	0.016	43.3	0.0029

### Contact distribution predictions of the model striatal networks

We used the expected intersection functions (equation 20) to construct model striatal networks, which we could examine for their predictions of striatal connectivity. We built networks within a 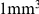 cube of striatal tissue, giving us 84900 MSNs, with 1%, 3% or 5% FSIs added (see Materials and Methods). For every neuron within 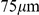 of the centre, we found all of its targets, afferents, and the distances to and from them. Restricting ourselves to this radius ensured that we could identify neurons that were little affected by their proximity to the edges of the volume, having complete afferent and efferent intra-striatal connectivity; hence we considered them the best candidates for comparing to, and making predictions about, the real striatum. To get sufficient numbers for analysis, we constructed 10 networks for each FSI percentage and pooled the data.

**Figure 8:**
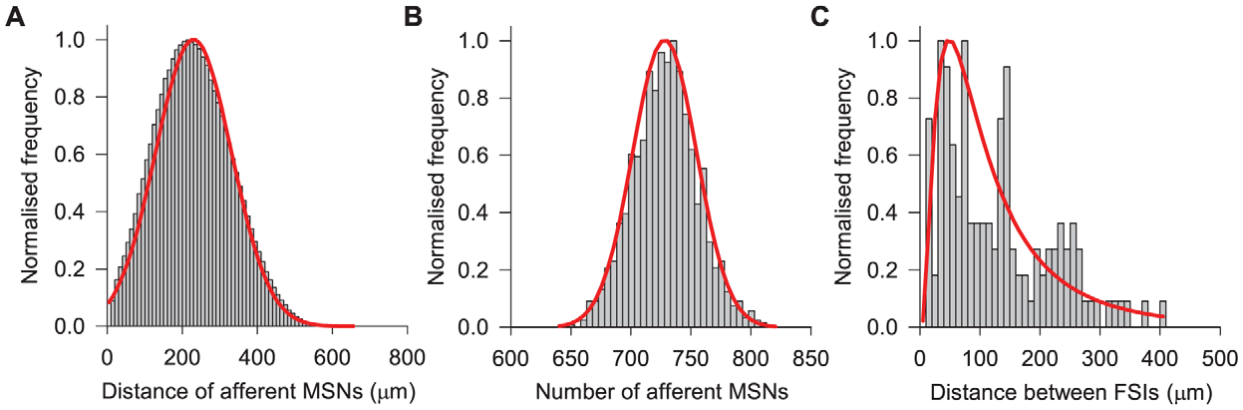
Model predictions for the statistics of striatal neuron connectivity. **A** Distribution of distances for MSN afferents to each MSN over all networks is approximately Gaussian (thick red line), truncated at the minimum enforced distance between neurons. **B** Distribution of number of afferent MSNs to each MSN is approximately Gaussian too. **C** The distances between gap-junction coupled FSIs follow a log-normal distribution, with half of all connections occurring between neurons less than 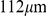 apart. (All data taken from 3% FSI networks. Histograms were compiled for 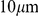 (panels A, C) or 5 neuron bins (panel B), and normalised to the maximum bin count.)

The constructed networks predict that, for all but the FSI gap junctions, the numbers of and distances between connected pairs of neurons have Gaussian distributions (Table 5), despite the complexity of the individual expected intersection functions (Figure 7). Figure 8 shows these Gaussian distributions for the MSN inputs to each MSN: each has 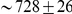 MSN afferents, at distances of 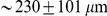 (note the distribution is truncated at the minimum distance of 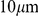). As we show in Figure 8C, the exception, consistent for each FSI percentage we tested, is the log-normal distribution of distances between gap-junction coupled FSIs.

**Table 5:** Connection statistics of the model striatal networks.

	Number of contacts			Distance ( 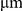 )		
	FSI 1%	FSI 3%	FSI 5%	FSI 1%	FSI 3%	FSI 5%
MSNs - 1 MSN	728 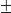 25.7	728 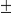 26.7	727 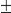 26.6	230 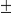 101	230 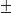 101	230 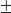 101
FSIs - 1 MSN	30.6 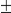 5.39	88.3 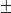 8.84	152 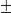 12.2	233 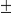 99.9	234 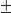 99.3	231 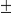 100
1 FSI - MSNs	3017 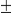 45.1	2992 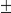 37.7	3011 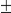 50.6	232 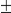 99.7	232 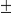 99.3	233 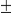 99.3
FSIs - 1 FSI	12.8 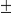 3.37	35.9 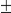 6.12	62.7 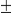 8.33	228 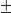 97	214 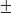 95.7	216 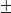 95.2
FSI gap	0.65 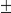 0.81	2.96 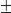 1.87	4.64 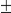 2.05	138 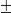 106	129 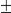 90	125 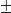 88.6
				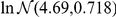	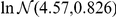	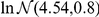

The first column names all the connection directions that can have distinct distributions of numbers of contacts and distances between connected pairs. For example, ‘MSNs - 1 MSN’ gives data for the numbers and distances of MSNs afferent to 1 MSN; conversely, ‘1 FSI – MSNs’ gives data for the numbers and distances of MSNs contacted by a single FSI. All values given as arithmetic mean 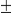 s.d., rounded to three significant figures. The second row for the FSI gap junction statistics gives the location 
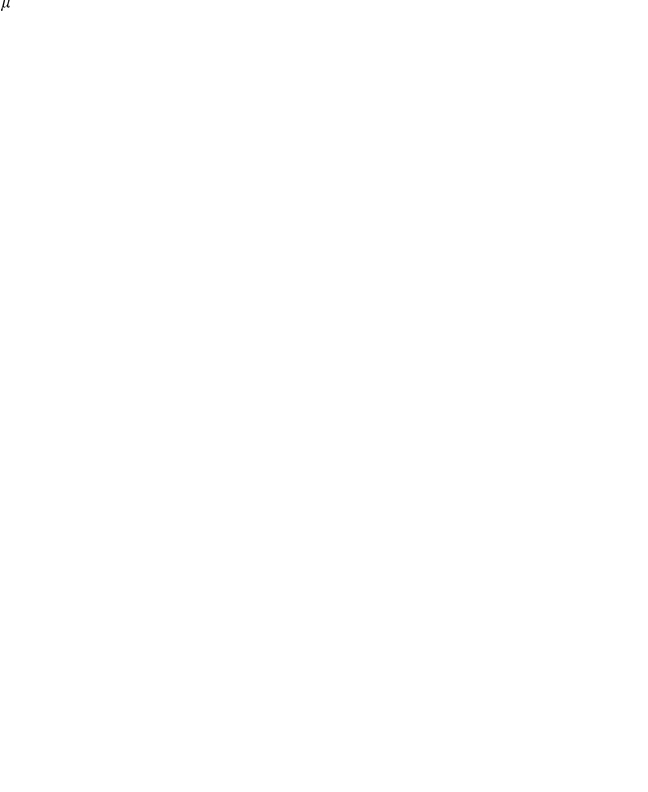
 and scale 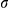 parameters for the best-fit log-normal distributions 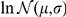.

Table 5 shows how the distributions of numbers and distances of contacts change for all connections across the 1, 3, and 5% FSI networks. The number of connected MSNs remains constant at around 728 MSNs afferent to one MSN. We found that if we restricted counting inter-connected MSNs to just those within 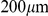 of each other, then each MSN receives 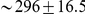 MSN afferents and, hence, has a 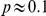 probability of being connected with another MSN in that radius, in excellent agreement with previous estimates (see *The microcircuit and connection statistics*). The number of MSNs contacted by one FSI (‘1 FSI-MSNs’ in Table 5) stayed constant, as expected, at around 3000 MSNs per FSI. The number of FSIs afferent to a single MSN increased with increasing FSI percentage. The 1% FSI network predicts around 30 FSIs per MSN, in good agreement with previous estimates of 4–27 FSIs per MSN [19], [44] – the other FSI percentage networks fall well outside these bounds. Similarly, the numbers of synaptic and gap junctions contacts between FSIs increased when increasing the percentage of FSIs in the network models. The mean numbers of gap junction contacts per FSI are only in good agreement with our estimated ranges from Fukuda's [39] data (see Table 1) for an FSI density of 1%.

A striking prediction of the network model is that the mean afferent distances for FSI and MSN inputs to a MSN and for FSI synaptic inputs to other FSIs are all 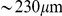 (for 1% FSI networks; the 3% and 5% networks have slightly lower mean distances for FSI input to other FSIs). This strongly suggests a natural spatial scale for the dominant inhibitory synaptic input to a MSN or FSI. Further, the network model predicts that a FSI's gap junction network is focussed locally around the neuron. Both these properties have implications for the dynamics of the striatum, which we illustrate below.

#### The sparseness of striatal connectivity

Here we illustrate that, despite the seemingly ‘large’ numbers of contacts for some connection types, the network predicts that connectivity is sparse. We compared our network results with a control model, which asked: what if each neuron contacted all others with which it shared an overlap of dendritic and axonal fields? Such a model would give numbers for a fully connected three-dimensional network, and provide a basis for understanding the sparseness of connectivity within the striatum. Following the numbers used in our full model, we assumed a MSN dendrite radius of 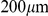, and FSI and MSN axonal field radii of 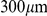. Similar to the full model, we constructed networks using 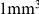 cubes of randomly positioned neurons, and counted all contacts for neurons within 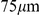 of the centre; except now we connected *all* MSN-MSN and FSI-MSN pairs whose axonal and dendritic fields overlapped. We found that the numbers of contacts in our network model (in Table 5) were consistently just 1.7% of all possible MSN-MSN contacts and 7% of all possible FSI-MSN contacts defined by this control model (irrespective of the FSI density used).

### Dynamical implications of network connectivity

The models of the striatal network revealed two striking features that could play a key role in striatal dynamics: the differing spatial scales for the inter-FSI gap junction and synaptic contact networks, and the common mean distances of GABAergic afferents to one MSN. We show here that both features indeed have the potential to set the input-output relationships of the striatum. To do so, we use a computational model of striatum that took the developed models of neurons (MSNs and FSIs), synapses (AMPA, NMDA, and GABAa) and gap junctions from our previous work [4], but used the striatal network model developed here as the basis for wiring the neurons together.

#### Effects of the spatial scales of inter-FSI networks

**Figure 9:**
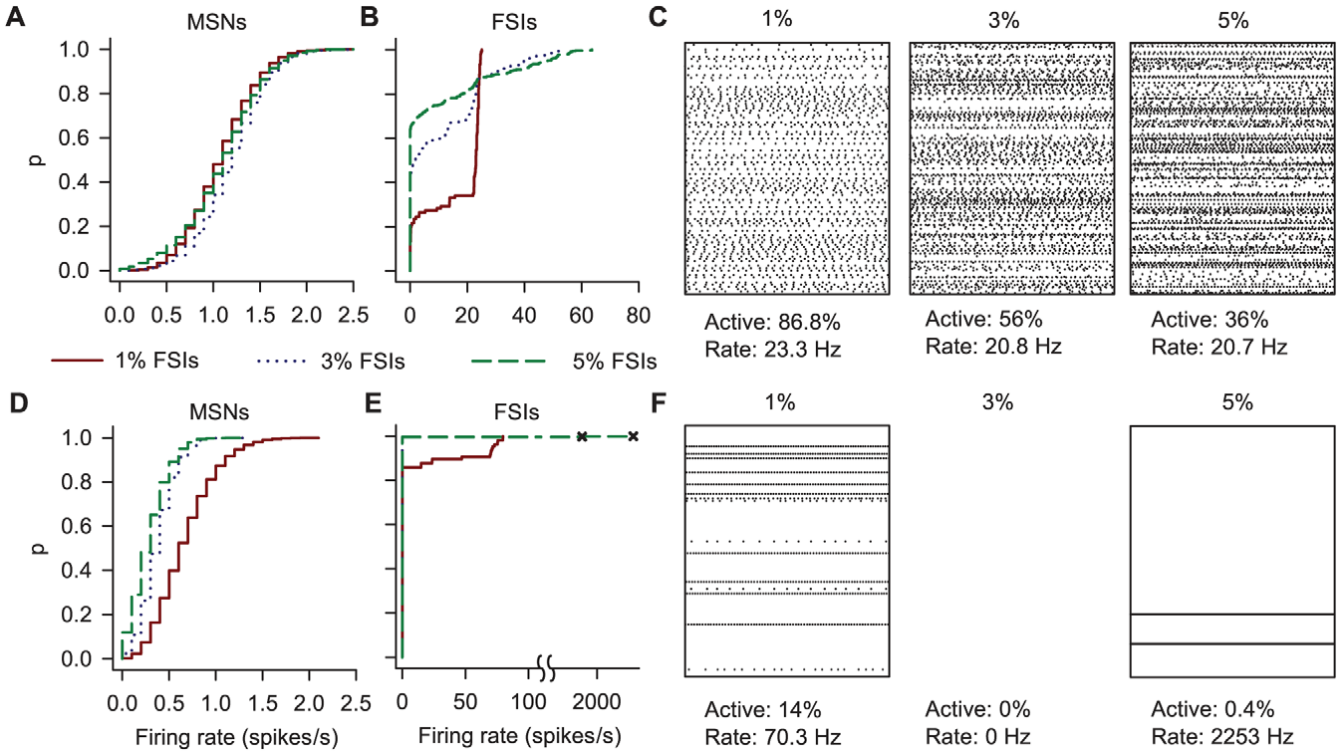
Effects on simulated striatal activity of changing the spatial scales of the inter-FSI synaptic and gap junction networks. We ran simulations of a 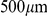-on-the-side cube of striatum (giving 10613 MSNs) for each FSI density (giving 106, 318, and 531 FSIs respectively); each neuron was driven for 10 simulated seconds by background cortical input of around 475 spikes/s – just above the threshold for causing a MSN to spike [80]. To investigate the effects of the spatial scales of inter-FSI connections, we ran two sets of simulations: one set (panels A–C) using networks built with the expected intersection functions reported here (equation 20 and Table 4); the other set (panels D–F) using networks built the same way except that the FSI-FSI gap junction and synaptic functions were swapped – thus inverting the spatial relationships between the inter-FSI gap junction and synaptic networks. **A** The resulting empirical cumulative distribution functions (ECDFs) of MSN firing rates for each density of FSIs when using the normal anatomical model. The distribution of MSN firing rates remained largely the same with increasing FSI density, and the model MSNs had very low firing rates, characteristic of MSN activity *in vivo*. **B** The resulting ECDFs of FSI firing rates from the same simulations, showing that increasing the density of FSIs increased the proportion of silent FSIs, but also broadened the distribution of firing rates. **C** Raster plots of 1 s of activity of all FSIs in each simulation, illustrating these changes in firing rate distribution: the figures given below each raster show how the median firing rate of the active FSIs remained relatively consistent, even though firing rate distributions broadened, and the proportion of active FSIs fell. **D** The ECDFs of MSN firing rates after swapping the FSI connection functions shows that the MSN firing rate distribution was no longer constant; indeed for 3% FSIs the distribution was that of an MSN-only model, as all FSIs were silent. **E** The corresponding ECDFs of FSI firing rates show a dramatic effect on FSI activity. For 1% FSIs, swapping the connection functions caused an increased proportion of silent FSIs, but with a broadened spread of rates compared to the normal model; the 3% FSI network was completely silent. The 5% FSI network entered a pathological state where only two FSIs fired at extreme rates (indicated by the two crosses). **F** These changes in FSI firing rate distribution are clear in the corresponding 1 s raster plots.

We first established the impact of the different FSI densities on the dynamics of the model. Figure 9 shows that increasing the FSI density did not alter the distribution of MSN firing rates or their variability (the median MSN inter-spike interval coefficient of variation was 0.8 for all FSI densities); nonetheless, the model MSNs had the very low firing rates characteristic of MSN activity *in vivo*. Increasing the FSI density increased the proportion of FSIs that did not fire, but also resulted in a broader and more heterogenous firing rate distribution. Despite this, the median firing rate of active FSIs was consistent across the changes in FSI density. The FSIs' firing rates of up to 80 spikes/s were also consistent with those observed *in vivo* [69]. Figure 9C shows that the active FSIs fired in a variety of desynchronised states, with no evidence of strong, network-wide synchrony for any tested FSI density.

We examined the effects of the different spatial scales of the inter-FSI gap junction and synaptic networks by then building models with swapped FSI-FSI gap junction and synaptic interconnection functions; hence, in these models, a FSI was synaptically coupled to FSIs close by, but gap-junction coupled to FSIs further away, inverting the spatial scales predicted by our network model. Figure 9D–F shows this produced a dramatic effect on striatal output. MSN population activity was now markedly affected by changes of FSI density. This correlated with the FSIs themselves becoming mostly (1%) or completely silent (3%), or entering a pathological state of two FSIs discharging at extreme rates (5%). The few active FSIs in the 1% FSI network tended to have higher firing rates than in the equivalent normal model. As the FSIs were completely silent in the 3% FSI network, the distribution of MSN activity in this case corresponds to a MSN-only network. Comparing this to all three distributions for the normal model in Figure 9A, we can see that the silencing of FSI activity caused a widespread decrease in MSN population activity.

#### Distance dependence of impact on MSN output

**Figure 10:**
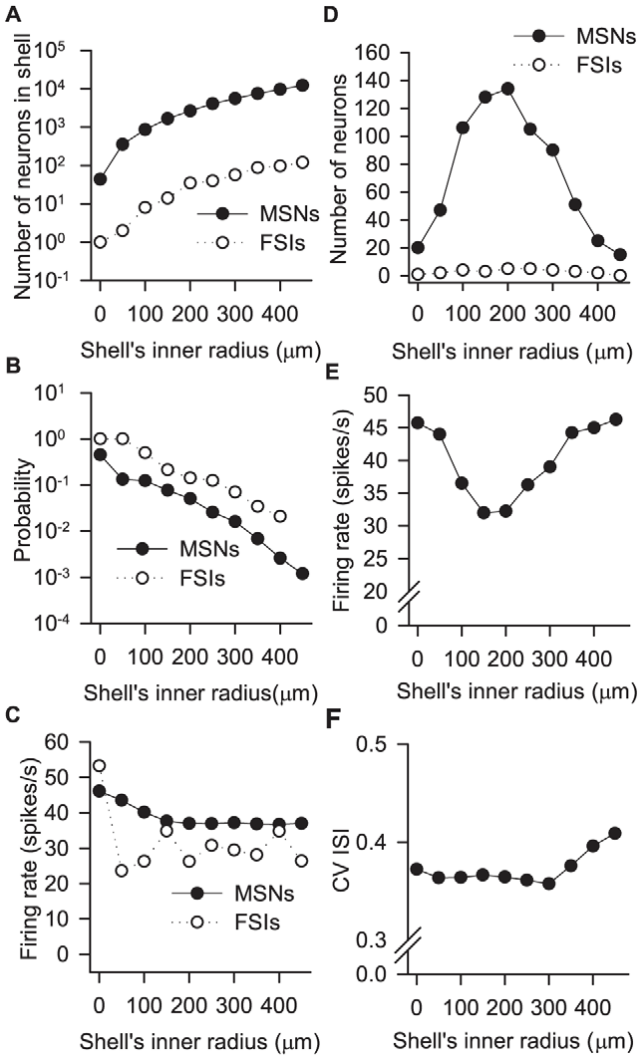
Implications of distance-dependent connections for MSN output. We stimulated all neurons within a 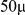
m wide spherical shell at varying distances from the centre of a 1mm-on-the-side cube of striatum (84900 MSNs, 1% FSIs) and studied the effect on the centre MSN's activity. **A** The total number of neurons per shell increases exponentially with increasing distance from the centre; here and in other panels we plot distances as the inner radius of the shell. **B** The probability of any chosen neuron in that shell contacting the central MSN falls exponentially with increasing distance. As contact probabilities are symmetric, this can also be read as approximately the distribution of probabilities for the central MSN contacting a given neuron in that shell. **C** All stimulated neurons received approximately 1250 spikes/s excitatory input for 4 seconds. The mean firing rate of MSNs in each shell fell slightly with increasing distance for the first few shells; the mean firing rate of FSIs in the shell was roughly constant (the first shell contained only one FSI). **D** The number of neurons projecting to the centre MSN peaked at the same distance for both MSN and FSI afferents, and confirm that the mean distances predicted by the network model (Table 5) do correspond to the distances of the greatest number of inputs. **E** In response to the same input as the stimulated neurons, the centre MSN's firing rate follows the inverse of the distribution of its inputs across the shells. **F** The centre MSN's inter-spike interval (ISI) coefficient of variation (CV), indicating the irregularity of the spike train, was also modulated by the distance of the afferent input.

The same mean distances of MSN and FSI afferents to one MSN imply that there will be a strong, non-monotonic, distance-dependent effect of those inputs on MSN activity. We examined this using a 1 mm-on-the-side cube of model striatum (84900 MSNs, and 1% FSIs) – a size chosen so that we could stimulate the inputs to the MSN nearest the centre of the cube by up to twice the mean distance of the afferents. To test the impact of input from a range of distances on the central MSN, we synaptically stimulated all the neurons in successive 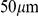-wide spherical shells around the central MSN (Figure 10A–C).

Figure 10D shows that the number of GABAergic inputs to the central MSN indeed peaks around the mean distance of FSI and MSN contacts at 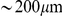. As a consequence, the central MSN's output was most strongly inhibited by inputs stimulated at this distance (Figure 10E). Across all stimulated shells, the central MSN's output was inversely correlated with the number of inputs at each distance (Figure 10E), but was not a function of the changes in firing rate in each shell's neurons (Figure 10C). We also observed that the distance of the inputs had a small modulatory affect on the regularity of the central MSN's spike-train (Figure 10F), but the relationship did not follow the same distance-dependent pattern. Figure S1 shows that all these effects, including the distance of maximum inhibition of the central MSN, are robust as they were the same even if we used an FSI density of 3%.

## Discussion

We have established a complete protocol for constructing a biologically-realistic network from first principles. The process described here is of general interest: in principle it could be used to model any region of the brain. It is particularly suited to the reconstruction of three-dimensional networks in non-layered structures, and we used it to reconstruct the GABAergic microcircuit of the adult rat striatum.

### Properties of dendrites, axons, and their intersections

Attempting to specify construction algorithms for the dendrites and axons showed specifically where quantitative morphology data were missing (we provide a complete list in Text S1). Building the MSN dendrite models revealed an inconsistency between previously reported total dendrite lengths and the number of spines on the MSN dendrites: the dendrite model had more wire, yet fewer spines. This suggests that the previously predicted range of 6250–15000 spines per MSN [68] is an overestimate: the model dendrites suggest a mean of 5932 spines per MSN – implying that, as each spine maintains a cortico-striatal synapse [40], [59], there are fewer cortical inputs to a MSN than previously estimated [68]. Moreover, the dendrite model predicts half of all spines are within 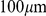 of the soma, half the radius of the MSN dendritic tree. As cortico-striatal synapses occur only on the spines [40], [59], this suggests half of all cortical input is to the proximal dendrites.

Using the axon and dendrite models, we found that achieving the target probabilities from [22] for MSN-MSN and FSI-MSN contacts within 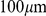 required large axon density constants 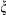. Matching the target MSN-MSN probability of 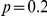 required an increase of the effective MSN axonal volume by a factor of 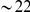; matching the target FSI-MSN probability of 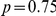 required an increase of the effective FSI axonal volume by a factor of 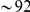. Both these results imply a dominant role for active processes guiding axon to dendrite in wiring up the striatum, beyond passive intersection of dendrite and axon alone. We used the same FSI axon scaling factor to construct the synaptic connections between FSIs: the intersection function for this connection is, hence, currently a prediction of the model. By contrast, we found that the density of FSI gap junctions was captured by the model using passive intersections of dendrites alone.

The expected number of intersections between neurons had a product-of-exponentials form (Figure 7), with five parameters whose precise values (in Table 4) would be difficult to recover from anatomical data. Nonetheless, the characteristic double-exponential function (equation 20) could, in principle, be recovered qualitatively. Furthermore, we have shown that the probability of contact between two neurons need not be a simple exponential function of distance [31].

### Statistics of striatal connectivity

When we applied the intersection functions to construct the striatal network models, we found though that almost all distributions of numbers of contacts and their distances were Gaussian. The network models predicted that each MSN receives an average of 728 inputs from other MSNs, when considering the complete network. Confidence in this result stems not just from the tuning to match the data on probability of contact within 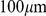, but also from the model having a mean number of 296 MSN-MSN contacts within 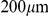, which is in excellent agreement with previous estimates (see *The microcircuit and connection statistics*). The network model predicts each FSI contacts around 3000 MSNs, which may explain why the FSIs, despite being few in number, are able to potently suppress MSN activity across the striatum [45].

The numbers of contacts for the other connection types were dependent on the percentage of FSIs in the network. Mean numbers of contacts in the 1% FSI network are consistent with existing estimates for the number of FSIs contacting one MSN [44], and the density of FSI-FSI gap junctions [39] (albeit at the lower end of the ranges we calculated from Fukuda's [39] data in Table 1). By contrast, the 3% and 5% FSI networks predict too many FSI inputs per MSN, and too many FSI gap junctions. Hence, the network model is consistent with recent estimates that at most 1% of striatal neurons are FSIs [19], [38], [39]. Given the decreasing density of FSIs over the dorsolateral-ventromedial axis of the striatum [12], [38], [70], it is plausible that even lower densities of FSIs occur in some striatal regions. Irrespective of the exact FSI percentage, the network models showed that the full three-dimensional network of the striatum is extremely sparse, forming around 1.7% of all possible MSN-MSN contacts and 7% of all possible single-FSI-to-many-MSNs contacts that could be made given the radii of dendritic and axonal fields.

The network models made two striking predictions about the spatial organisation of contacts in the striatum. First, that the networks of gap junctions and synapses inter-connecting FSIs were on different spatial-scales: the log-normal, left-skewed distribution of gap junction distances implies each FSI makes most of its electrical connections with immediately neighbouring FSIs; the Gaussian distribution of synaptic distances implies each FSI makes most of its synaptic connections with FSIs more distally. Second, that inputs to a MSN from either FSIs or other MSNs are on the same length scale of 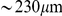. This result illustrates the unintuitive nature of three-dimensional connectivity: the fall-off in the probability of connection is counteracted by an increase in the number of neurons to make connections with, so that the dominant distance of connections is some function of both.

### Implications for the dynamics of the striatum

The anatomical model results point to some intriguing implications for the spatial scales of computation in the striatum. The “domain” theory [9], [24], [25] suggests that the natural computational element of the striatum is the network of MSNs within the 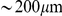 radius of one MSN's dendritic field. On the one hand, the network models confirm that MSNs have an approximately 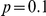 probability of contacting another MSN within that radius, far greater than the probability of contacting farther MSNs, suggesting the formation of a closely-knit network – if we consider only probability of connection. On the other hand, our network models show us that the number of connections give us the inverse of the domain concept: a MSN receives its greatest number of inputs from MSNs and FSIs whose soma lie just on edge of the main extent of its dendrites. The comparatively weak nature of the individual MSN-MSN synapse – with a mean conductance approximately five times smaller than the FSI-MSN synapse [21], [22], [44] – suggests that the number of MSN inputs is the key factor in understanding the influence of the local MSN axon collateral network. We showed in a computational model that this is indeed the case: the most potent inhibition of an active MSN was achieved by stimulating inputs around 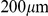 away (Figure 10E, Figure S1). Therefore, if a ‘computational element’ of the striatum is defined by the spatial scales of feedback and feedforward inhibition – from other MSNs and FSIs, respectively – then our models show it to be spread over the network, not concentrated locally within the dendritic field.

The spontaneous organisation of activity in striatum is consistent with such a widespread network of effective MSN-MSN connections. Carillo-Reid et al [29] showed that global excitation *in vitro* induced the appearance of a few cell assemblies within an 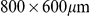 plane, with each assembly comprising neurons spread over the plane. Models of this phenomenon from both us [4] – using distance-dependent connections, as here – and Ponzi and Wickens [16] – using uniform probability of connection – show that such cell assemblies are not formed by discrete groups in physical space. The data and models also showed that such assemblies contain comparatively small numbers of neurons (at most a few hundred) on the scale of other definitions for a striatal ‘computational-element’. Future work with the model reported here will examine the reasons for this discrepancy. What the network model, and the computational model built upon it, do make clear is that further understanding the computations performed by the striatal microcircuit requires better knowledge of the distribution of individual cortical inputs [63], [68], to understand if they are organised along any of the characteristic spatial scales of the striatal network.

The network model also showed that the density of FSIs affects both the number and spatial-scales of connections. We showed that these anatomical effects are reflected in changing dynamical properties of the FSI network in the computational striatum model. Changing the FSI density altered the distribution of FSI firing rates, decreasing the proportion of active FSIs, but increasing the range of rates. However, irrespective of the FSI density, the FSI network remained in a globally-asynchronous state, with many FSIs completely or nearly silent. Though contrary to previous reports that networks of spiking neurons coupled by both gap-junctions and inhibitory synapses promote globally synchronised activity (e.g. [71]), our findings are consistent with both our previous work [4], and with Lau et al's [15] report that asynchronous, partially-silent states dominate in such networks if the gap junction network is not wired together as a classic random network – that is, one with a uniform probability of connection. In the case of Lau et al [15], this wiring was a small-world network on a ring-lattice; here our striatal anatomy model deviates from a classic random network because of the distance-dependent probability of the gap junction connections between the FSIs. Taken together, Lau et al's [15] results, and ours here and previously [4], all point to the importance of considering both the wiring topology and its spatial embedding when considering the dynamics – and, hence, likely function – of interneurons coupled by both gap junctions and inhibitory synapses.

The local and distal networks formed respectively by the inter-FSI gap junctions and synapses produced characteristic properties of the MSN population dynamics too. The MSN population activity was remarkably consistent across changes in FSI density (Figure 9A), despite the changes in FSI activity just described. However, when we swapped the inter-FSI networks (gap junctions distally, synapses locally), the MSN firing rates now changed with changing FSI density. Hence, the combination of local gap junction and distal synaptic networks predicted by the model constrains the whole MSN population to a particular input-output regime, robust to changes in FSI density.

We also saw that in the absence of active FSIs, the MSN population activity was globally reduced compared to all normal models with active FSIs. This result in a larger and anatomically more detailed model confirms our previous finding that removing all FSIs reduces model MSN activity [4]. The unintuitive effect that increasing the number of GABAergic interneurons increases the firing rate of their target neurons has a clear underlying cause: the GABA reversal potential is above the typical MSN ‘down’-state membrane potential [11], and hence sporadic FSI input to MSNs will tend to keep their membrane potential relatively depolarised, allowing them to fire (and fire more often) to excitatory input (too much FSI input, however, would clamp the MSN membrane potential at the GABA reversal potential).

### Applications, extensions, omissions

The results of this work have further applications in the study of both single-neuron and network-level dynamics. By using our found parameters for the Burke algorithm, it is possible to generate many MSN and FSI dendritic morphologies, each consistent with current morphological data. Hence, instantiating the same multi-compartmental model (e.g. [13], [67], [72]) on multiple instances of these generated morphologies will open up a wide range of applications, such as placing limits on post-synaptic potential summation, back-propagating action potentials, maximal conductance searches, and so on. At the network level we have shown how we gain the benefits of reconstructing the underlying structure, as argued at the outset of this paper. Particularly interesting will be the results of using these reconstructed networks – requiring only equation (20) – as the basis for other group's approaches to modelling the striatum [13]–[16], [72], [73].

We have focussed on the principal GABAergic microcircuit of the striatum here, as this provides the basis for the most immediate, powerful control over the output of the striatum [4], [45], [50]. The current work has thus omitted other interneuron types. A full striatal network reconstruction would include the giant cholinergic interneurons with their dense and long-reaching axonal ramifications that synapse on MSNs [37], [74], and the low-threshold spiking interneurons [37], which may form an inhibitory network between the cholinergic interneurons [75] or control gap junction efficacy through the release of nitric oxide [21], [76]. In addition, the network construction does not currently address what happens at the histochemically defined borders between the ‘patch’ and ‘matrix’ of the striatum [34], which many MSN dendrites do not cross [77].

Our model is a stochastic realisation of the adult striatum; the modelling of developing striatal connectivity is a stern challenge given the current paucity of data [78]. Nonetheless, we think modelling the development of connectivity is essential to capture elements of striatal wiring we have not accounted for in the present model. For example, recent work on BAC transgenic mice suggests a preferential direction of connection between the two populations of MSNs defined by their dominant dopamine receptor type (D1 or D2), with significantly fewer projections from D1-expressing to D2-expressing MSNs than any other combination [21], [22], [79]. With no data yet on how such selective connectivity might form, we must chalk this up as a future target for our models. Clearly we are only at the beginning of constructing realistic models of the striatum; but equally we have a promising start.

## Supporting Information

Figure S1 Implications of distance-dependent connections for MSN output. We stimulated all neurons within a 50 µm wide spherical shell at varying distances from the centre of a 1mm-on-the-side cube of striatum (84900 MSNs). Here we used a network with a FSI density of 3% (2547 FSIs) to check that the effects on the centre MSN were consistent even with increasing numbers of FSIs. A. The total number of neurons per shell increases exponentially with increasing distance from the centre; here and in all other panels we plot distances as the inner radius of the shell. B. The probability of any chosen neuron in that shell contacting the central MSN falls exponentially with increasing distance. C. All stimulated neurons received approximately 1250 spikes/s excitatory input for 4 seconds. The mean firing rate of MSNs in the shell fell slightly with increasing distance; the mean firing rate of FSIs in the shell was roughly constant (the first shell contained only one FSI). D. The number of neurons projecting to the centre MSN peaked at the same distance for both MSN and FSI afferents. E. In response to the same input as the stimulated neurons, the centre MSN's firing rate follows the inverse of the distribution of inputs across the shells. F. The centre MSN's inter-spike interval (ISI) coefficient of variation (CV), indicating the irregularity of the spike train, was more modulated by the distance of the afferent input than for the 1% FSI network.
(0.19 MB PDF)

Text S1 Specification of the Burke algorithm and the evolutionary algorithm used for searching the Burke algorithm parameter space; also includes a list of missing/desired morphological data.
(0.12 MB PDF)
