# Supplementary material for: Reconstructing the Three-Dimensional GABAergic Microcircuit of the Striatum
Source: PLoS Comput Biol. 2010 Nov 24;6(11):e1001011. doi: 10.1371/journal.pcbi.1001011 (PMC2991252; doi:10.1371/journal.pcbi.1001011)
Supplement: Figure S1 — Implications of distance-dependent connections for MSN output. We stimulated all neurons within a 50 µm wide spherical shell at varying distances from the centre of a 1mm-on-the-side cube of striatum (84900 MSNs). Here we used a network with a FSI density of 3% (2547 FSIs) to check that the effects on the centre MSN were consistent even with increasing numbers of FSIs. A. The total number of neurons per shell increases exponentially with increasing distance from the centre; here and in all other panels we plot distances as the inner radius of the shell. B. The probability of any chosen neuron in that shell contacting the central MSN falls exponentially with increasing distance. C. All stimulated neurons received approximately 1250 spikes/s excitatory input for 4 seconds. The mean firing rate of MSNs in the shell fell slightly with increasing distance; the mean firing rate of FSIs in the shell was roughly constant (the first shell contained only one FSI). D. The number of neurons projecting to the centre MSN peaked at the same distance for both MSN and FSI afferents. E. In response to the same input as the stimulated neurons, the centre MSN's firing rate follows the inverse of the distribution of inputs across the shells. F. The centre MSN's inter-spike interval (ISI) coefficient of variation (CV), indicating the irregularity of the spike train, was more modulated by the distance of the afferent input than for the 1% FSI network. (0.19 MB PDF) [file pcbi.1001011.s001.pdf]

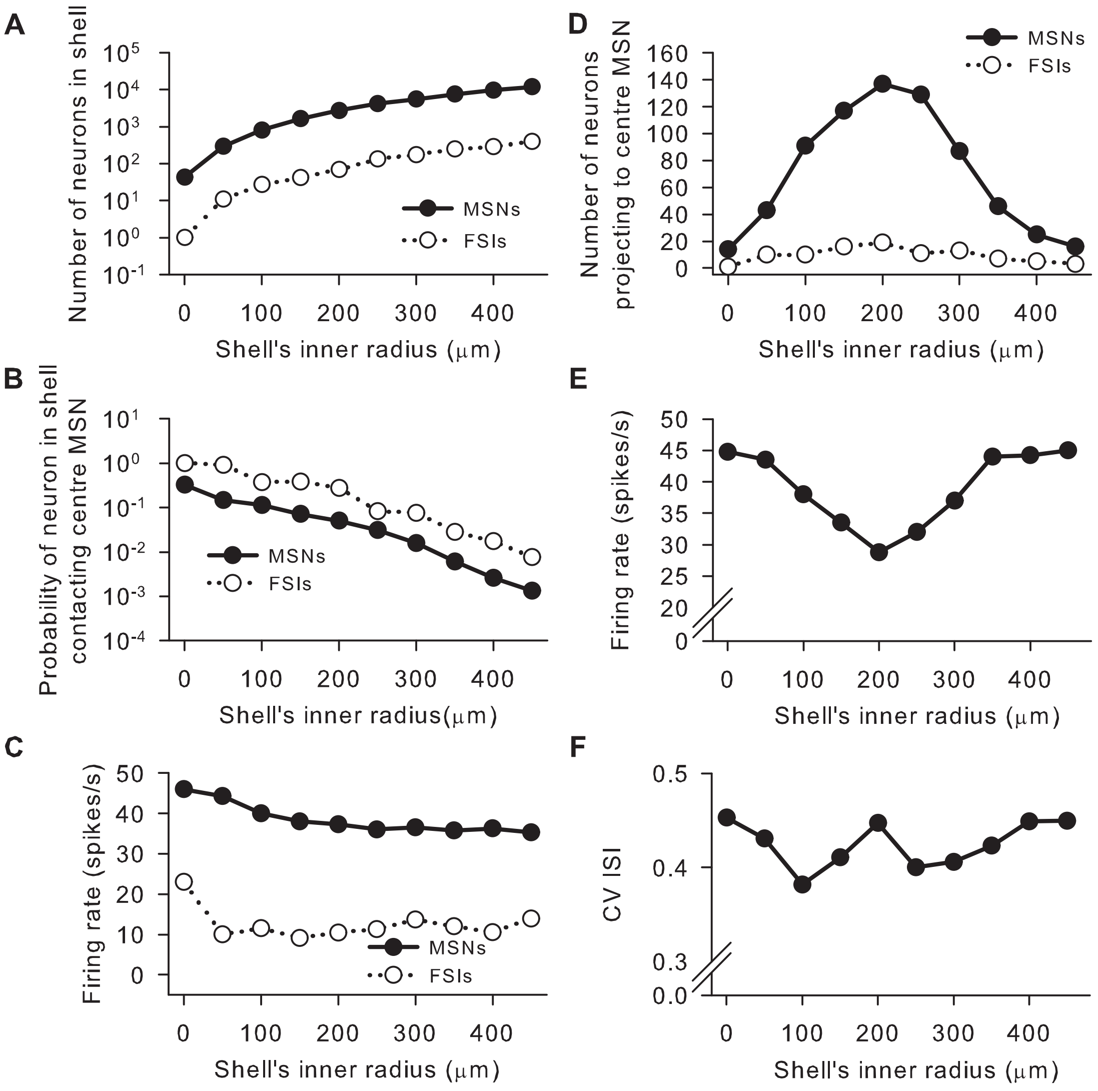

Figure S1. Implications of distance-dependent connections for MSN output. We stimulated all neurons within a 50μm wide spherical shell at varying distances from the centre of a 1mm-on-the-side cube of striatum (84900 MSNs). Here we used a network with an FSI density of 3% (2547 FSIs) to check that the effects on the centre MSN were consistent even with increasing numbers of FSIs. **A** The total number of neurons per shell increases exponentially with increasing distance from the centre; here and in all other panels we plot distances as the inner radius of the shell. **B** The probability of any chosen neuron in that shell contacting the central MSN falls exponentially with increasing distance. **C** All stimulated neurons received approximately 1250 spikes/s excitatory input for 4 seconds. The mean firing rate of MSNs in the shell fell slightly with increasing distance; the mean firing rate of FSIs in the shell was roughly constant (the first shell contained only one FSI). **D** The number of neurons projecting to the centre MSN peaked at the same distance for both MSN and FSI afferents. **E** In response to the same input as the stimulated neurons, the centre MSN's firing rate follows the inverse of the distribution of inputs across the shells. **F** The centre MSN's inter-spike interval (ISI) coefficient of variation (CV), indicating the irregularity of the spike train, was more modulated by the distance of the afferent input than for the 1% FSI network.
