# Supplementary material for: Reconstructing the Three-Dimensional GABAergic Microcircuit of the Striatum
Source: PLoS Comput Biol. 2010 Nov 24;6(11):e1001011. doi: 10.1371/journal.pcbi.1001011 (PMC2991252; doi:10.1371/journal.pcbi.1001011)
Supplement: Text S1 — Specification of the Burke algorithm and the evolutionary algorithm used for searching the Burke algorithm parameter space; also includes a list of missing/desired morphological data. (0.12 MB PDF) [file pcbi.1001011.s002.pdf]

Supporting Information for: Reconstructing the three dimensional GABAergic microcircuit of the striatum

Mark D. Humphries, Ric Wood, Kevin Gurney

The Burke algorithm for constructing dendrite models

The Burke algorithm has two probability functions based on the diameter θ of the current dendritic segment: the probability of terminating the current branch $p(T|\theta)$; and the probability of branching $p(B|\theta)$, which is obtained by evaluating two subsidiary distributions and using the minimum value: $p(B|\theta) = \min\{p_1(B|\theta), p_2(B|\theta)\}$.

The form of the algorithm we use for constructing a single dendritic tree is as follows

1. Start with an initial dendritic segment with length ΔL and diameter θ_0.
2. For each unterminated segment:
   (a) Generate a uniformly distributed random number $R \in [0,1]$
   (b) If $R \in [0, p(T|\theta)\Delta L]$ then terminate the branch, return to the beginning of step 2.
   (c) If $R \in (p(T|\theta)\Delta L, (p(T|\theta) + p(B|\theta))\Delta L]$ then the segment ends in a branch point, and two new branches are generated with lengths of ΔL, and diameters, θ_1 and θ_2, of

   $$\theta_1 = \theta_{\text{par}}(r_1 + ar_2),$$
   $$\theta_2 = \theta_{\text{par}}(r_2 + ar_1),$$

   where θ_{par} is the diameter of the parent segment, a is a constant, and r_1 and r_2 are drawn from a Gaussian distribution $G(\mu_{\text{r}}, \sigma_{\text{r}})$.
   (d) Otherwise a new segment is added to the dendrite, with length ΔL and diameter $(1 - t\Delta L)\theta_{\text{par}}$, where t sets the tapering rate of the dendrite. A minimum diameter θ_{min} is enforced, beyond which the dendrite cannot taper further.
3. Repeat from the beginning of step 2 until all segments have terminated.

We repeat this for each tree required to construct a complete dendrogram for the neuron.

Morphological data

As for most neuron types, data on the diameters of dendrites at branch and termination points are not available for MSNs and FSIs, and so we could not apply the Burke algorithm directly. Instead, we gathered morphological data on the known properties of their dendritic trees, then searched to find the Burke algorithm parameters that result in dendrograms fitting the gathered data.

The target data to constrain the search was found for branch order, dendritic radius, number of terminals, and terminal diameter (Table S1). Quantitative data on striatal FSI morphology is lacking, and so we used data on branch order and number of terminals from studies of cortical FSIs (specifically basket cells). The strong physiological and morphological similarities between the two classes [1,2], with the exception of their axon trajectories, makes this a reasonable substitute in the absence of specific data.

We also required the number of dendritic trees to know how many times to repeat the Burke algorithm to get a complete dendrogram, and the initial diameters θ_0 to supply to the algorithm. For a MSN dendrogram we constructed 6 trees (range 4-8 trees, [3,8]; or 3-11 trees, [6]), each with an initial diameter of $\theta_0 = 2\mu$m (range $0.5 - 2.5\mu$m [6,8]). For a FSI dendrogram we constructed 5 trees (range 3-8, [12]), each with an initial diameter of $\theta_0 = 1.5\mu$m [11]. To constrain the Burke algorithm, we also noted that the maximum reported radii for the

Table S1. Bounds placed on dendrogram properties. All data obtained from the rat. Entries marked with * indicate data only available from cortical FSIs.

Neuron	Property	Data	References	Bounds used in search
MSN	Maximum branch order	5	[3,4]	$[0,5]$
	Mean number of terminals	~ 27	[3,5,6]	$[25,35]$
	Mean terminal diameter	0.29μm	[7]	$[0.25,0.45]\mu$m
	Mean dendritic radius	$\sim 200\mu$m	[3,5,8]	$[100,350]\mu$m
FSI	Maximum branch order*	4	[9]	$[0,4]$
	Number of terminals*	12-16	[10]	$[9,19]$
	Mean terminal diameter	0.4μm	[11]	$[0.2,0.4]\mu$m
	Mean dendritic radius	$100-200\mu$m	[12]	$[100,250]\mu$m

dendrites are respectively $\sim 300\mu$m [3] and $\sim 200\mu$m [12–14] for MSNs and FSIs, and that the MSNs have a maximum of 81 branch points [6] — no data on branch points are available for FSIs.

The other parameters for the Burke algorithm were set to the following values. We used a step of $\Delta L = 1\mu$m. Taper rate was $t = 0.005$. The minimum diameter was set to $\theta_{\text{min}} = 0.2\mu$m. Other parameter values were taken from [15], as no other data were available, hence: $a = -0.2087$, $\mu_{\text{r}} = 0.862$, $\sigma_{\text{r}} = 0.213$.

Construction of the evolutionary algorithm search

We used an evolutionary algorithm search to find two sets of parameter values for the Burke algorithm, one for MSN and one for FSI dendrograms. Each candidate in the search comprised the 6 parameters of the probability functions, namely k_1 and k_2 for each of $p_1(B|\theta)$, $p_2(B|\theta)$ and $p(T|\theta)$. The general form of the search is as follows:

1. Create an initial population of n_0 candidates, each element of a candidate initialised within the defined range for its corresponding parameter (see below).

2. For each member of the population, repeat the following m times

   (a) Check that the element values result in valid probability distributions. First, that $\forall \theta \in [\theta_{\text{min}}, \theta_0]$, $[p(T|\theta) + p(B|\theta)]\,\Delta L \leq 1$. Second, that the slope of the branching probability function is greater than zero, specifically that $p(B|\theta_{\text{max}}) - p(B|\theta_{\text{min}}) > 0.01$. If either of these checks fails, then the candidate is deemed a failure and the process returns to the beginning of step 2.

   (b) Run the Burke algorithm using the element values, generating the appropriate number of dendritic trees for the neuron class. Two running checks are made. First, that the total length of a single dendritic tree has not exceeded some value l_{max}. Second, that the total number of branch points, summed over the whole dendrogram, has not exceeded some value b_{max}. (These are necessary because the Burke algorithm can run indefinitely for some combinations of parameters). If either of these checks fails, then the candidate is deemed a failure and the process returns to the beginning of step 2.

   (c) The final dendrogram produced by the Burke algorithm for those element values is evaluated against the appropriate bounds on the morphological properties given in Table 1 (main text). If any bound is exceeded, then the candidate is deemed a failure; otherwise it is a success.

3. The fitness of each candidate is then: (number of successes)$/m$.

4. If fitness is maximal (i.e. is m) or the number of generations is greater than G_{max}, then the search is terminated.

5. Otherwise, the population is ranked by fitness and a new population of size n is formed by mating and mutation of the most successful candidates (details given below) — return to the beginning of step 2.

All of our searches had the following properties. The initial population had $n_0 = 500$ members, and the 6 elements of each candidate were initialised with a random value within the corresponding parameter bounds in Table S2. All subsequent generations had populations of $n = 100$ members. The maximum number of generations was $G_{\max} = 600$. Each member was evaluated $m = 30$ times. From each generation, the top 75 ranked members were retained as the parent population: 25 new offspring were created by single-point crossover between candidate pairs selected uniformly at random from the parent population. Every member of the parent population — other than the most fit candidate — was subject to mutation with a probability of 0.05 per element; if selected for mutation, an element was altered to a real number within the parameter bounds from Table S2.

The morphological parameters required for the search were set as follows. The maximum diameter (for function checks in step 2(a) above) was $\theta_{\max} = 3\mu$m. The values for the running checks on the algorithm (step 2(b) above) were set to considerably exceed the largest reported value in the literature (see *Morphological data* above): hence, for MSNs, $l_{\max} = 1000\mu$m and $b_{\max} = 100$; for FSIs $l_{\max} = 750\mu$m and $b_{\max} = 100$.

Table S2. Bounds for the Burke algorithm parameters used in the evolutionary algorithm search.

	k_1	k_2
$p_1(B\|\theta)$	$[0.005, 0.1]$	$[10, 100]$
$p_2(B\|\theta)$	$[0.0005, 0.1]$	$[0.05, 1]$
$p(T\|\theta)$	$[0.05, 1]$	$[-1-20]$

Missing morphological data

The basic approach used here can be re-applied every time any of the missing data is supplied. The (incomplete) ambitious list of this missing data is as follows.

- Branch and termination diameters for either MSNs or FSIs would allow us to apply the Burke algorithm directly. Moreover, with that data available, we would be in a stronger position to use the second Burke algorithm, as we would then have fewer free parameters to account for.
- The relative change in diameter of each daughter branch compared to its parent branch (Burke algorithm parameters μ_r and σ_r).
- The relative diameters of each daughter branch compared to the other (Burke algorithm parameter a).
- Data on maximum branch order and number of terminal branches for striatal FSIs, as we have sourced this data from cortical FSIs here.
- Any quantitative data on MSN axons that would allow either a more accurate form of Equation 3 (main text) or, better still, a stochastic construction model akin to the Burke algorithm.
- Any quantitative data on the striatal FSI axons.
- Data on synapse distributions on MSN dendrites. This would allow us make a quantitative approximation to the specific targets of the MSN and FSI axon collaterals. Strangely, some of this data is available for striatal FSIs [11] despite the paucity of data on these neurons in other areas.

References

1. Kawaguchi Y (2003) Local circuit neurons in the frontal cortico-striatal system. In: Hensch TK, Fagiolini M, editors, Excitatory-Inhibitory Balance: Synapses, Circuits, Systems, Springer. pp. 125-148.

2. Kawaguchi Y, Karube F, Kubota Y (2006) Dendritic branch typing and spine expression patterns in cortical nonpyramidal cells. Cereb Cortex 16: 696–711.

3. Wilson CJ, Groves PM (1980) Fine structure and synaptic connections of the common spiny neuron of the rat neostriatum: a study employing intracellular inject of horseradish peroxidase. J Comp Neurol 194: 599–615.

4. Flores G, Alquicer G, Silva-Gomez AB, Zaldivar G, Stewart J, et al. (2005) Alterations in dendritic morphology of prefrontal cortical and nucleus accumbens neurons in post-pubertal rats after neonatal excitotoxic lesions of the ventral hippocampus. Neuroscience 133: 463–470.

5. Kawaguchi Y, Wilson CJ, Emson PC (1990) Projection subtypes of rat neostriatal matrix cells revealed by intracellular injection of biocytin. J Neurosci 10: 3421–3438.

6. Meredith GE, Agolia R, Arts MP, Groenewegen HJ, Zahm DS (1992) Morphological differences between projection neurons of the core and shell in the nucleus accumbens of the rat. Neuroscience 50: 149–162.

7. Wilson CJ (1992) Dendritic morphology, inward rectification and the functional properties of neostriatal neurons. In: McKenna T, Davis J, Zornetzer SF, editors, Single Neuron Computation, San Diego: Academic Press. pp. 141–171.

8. Preston RJ, Bishop GA, Kitai ST (1980) Medium spiny neuron projection from the rat striatum: an intracellular horseradish peroxidase study. Brain Res 183: 253–263.

9. Klostermann O, Wahle P (1999) Patterns of spontaneous activity and morphology of interneuron types in organotypic cortex and thalamus-cortex cultures. Neuroscience 92: 1243–1259.

10. Parnavelas JG, Uylings HB (1980) The growth of non-pyramidal neurons in the visual cortex of the rat: a morphometric study. Brain Res 193: 373–382.

11. Kita H, Kosaka T, Heizmann CW (1990) Parvalbumin-immunoreactive neurons in the rat neostriatum: a light and electron microscopic study. Brain Res 536: 1–15.

12. Kawaguchi Y (1993) Physiological, morphological, and histochemical characterization of three classes of interneurons in rat neostriatum. J Neurosci 13: 4908–4923.

13. Koos T, Tepper JM (1999) Inhibitory control of neostriatal projection neurons by GABAergic interneurons. Nat Neurosci 2: 467–472.

14. Kubota Y, Kawaguchi Y (2000) Dependence of GABAergic synaptic areas on the interneuron type and target size. J Neurosci 20: 375–386.

15. Burke R, Marks W, Ulfhake B (1992) A parsimonious description of motoneuron dendritic morphology using computer simulation. J Neurosci 12: 2403–2416.
